# New compounds identified through *in silico* approaches reduce the α-synuclein expression by inhibiting prolyl oligopeptidase *in vitro*

**DOI:** 10.1038/s41598-017-11302-0

**Published:** 2017-09-07

**Authors:** Raj Kumar, Rohit Bavi, Min Gi Jo, Venkatesh Arulalapperumal, Ayoung Baek, Shailima Rampogu, Myeong Ok Kim, Keun Woo Lee

**Affiliations:** Division of Applied Life Science (BK21 Plus), Systems and Synthetic Agrobiotech Center (SSAC), Plant Molecular Biology and Biotechnology Research Center (PMBBRC), Research Institute of Natural Science (RINS), Gyeongsang National University (GNU), 501 Jinju-daero, Jinju, 52828 Republic of Korea

## Abstract

Prolyl oligopeptidase (POP) is a serine protease that is responsible for the maturation and degradation of short neuropeptides and peptide hormones. The inhibition of POP has been demonstrated in the treatment of α-synucleinopathies and several neurological conditions. Therefore, ligand-based and structure-based pharmacophore models were generated and validated in order to identify potent POP inhibitors. Pharmacophore-based and docking-based virtual screening of a drug-like database resulted in 20 compounds. The *in vitro* POP assays indicated that the top scoring compounds obtained from virtual screening, Hit 1 and Hit 2 inhibit POP activity at a wide range of concentrations from 0.1 to 10 µM. Moreover, treatment of the hit compounds significantly reduced the α-synuclein expression in SH-SY5Y human neuroblastoma cells, that is implicated in Parkinson’s disease. Binding modes of Hit 1 and Hit 2 compounds were explored through molecular dynamics simulations. A detailed investigation of the binding interactions revealed that the hit compounds exhibited hydrogen bond interactions with important active site residues and greater electrostatic and hydrophobic interactions compared to those of the reference inhibitors. Finally, our findings indicated the potential of the identified compounds for the treatment of synucleinopathies and CNS related disorders.

## Introduction

**Figure 1:**
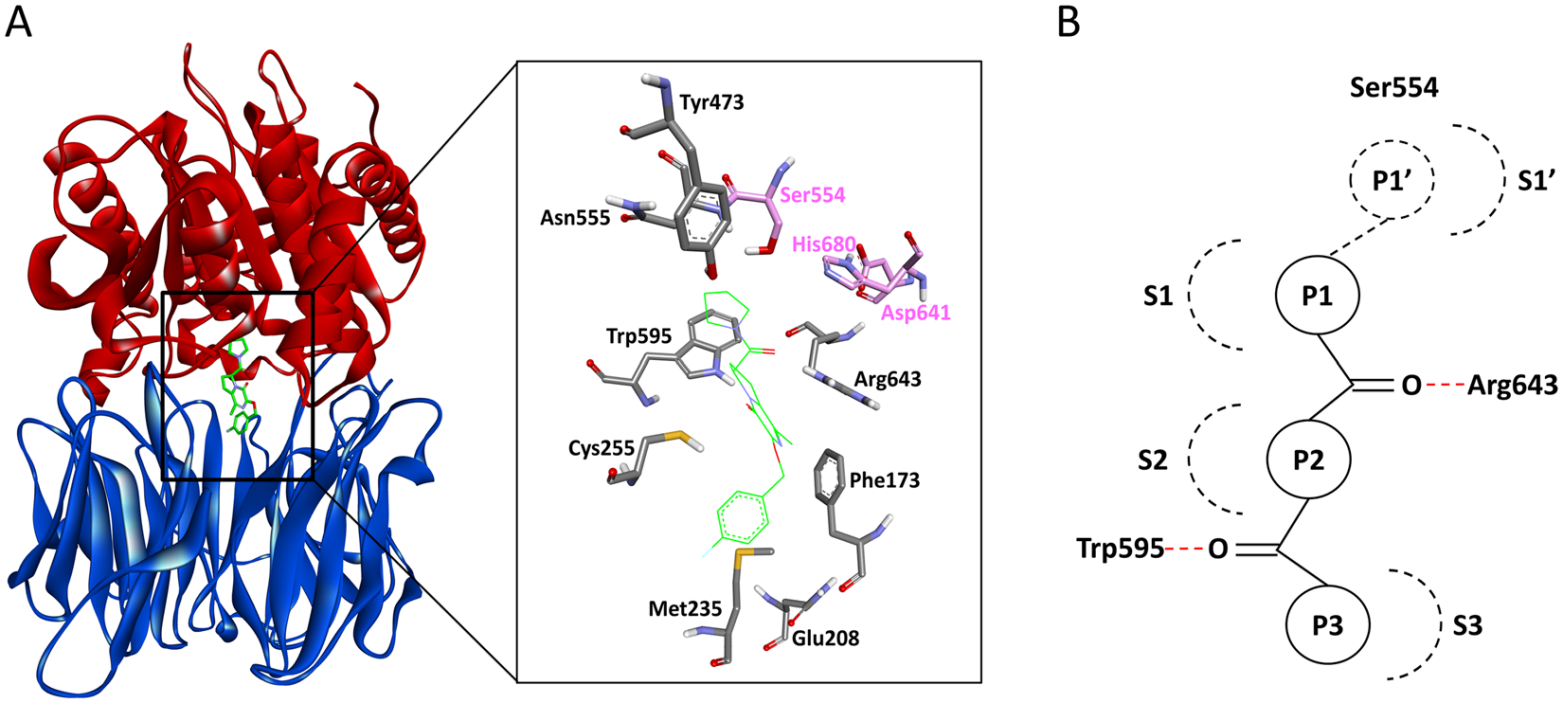
The structure of the human prolyl oligopeptidase (PDB ID: 3DDU) with functional subunits designated by colour. (**A**) The protease catalytic domain is shown in red, the β-propeller domain is shown in blue and the bound inhibitor is shown in green. The active site residues are represented in a stick model, and the catalytic residues are highlighted in a pink colour. (**B**) The general scheme of the pharmacophores showing the complementary interaction sites (Sx) at the POP active site with portions (Px) of the substrate or peptidomimetic inhibitors^24^. The dotted red lines represent the hydrogen bonds.

Prolyl oligopeptidase (POP) (PREP or Prolyl endopeptidase; EC 3.4.21.26) belongs to the serine protease family. POP is prominently expressed in various parts of the human and rodent brain, including the hypothalamus, hippocampus, cortex, striatum, and amygdala^1, 2^. POP cleaves the carboxyl side of proline residues of peptides that are shorter than 30 amino acids and is believed to play a role in the maturation and degradation of neuropeptides and peptide hormones^3–5^. Additionally, POP is also capable of metabolizing the oncolytic or cytotoxic agent tasidotin. However, the importance of POP in relation to cytotoxicity is currently unexplored^6^. The most important substrates of POP involve several biologically active peptides, such as angiotensins I and II, substance P, thyrotropin releasing hormone, arginine vasopressin, bradykinin, and oxytocin^7, 8^. In addition to its hydrolytic activity, POP has been reported to be involved in inositol-1,4,5-trisphosphate (IP3) signalling^9, 10^, the regulation of intracellular trafficking and protein secretion^11^, and nerve cell growth through protein–protein interactions^12^. Moreover, POP correlates with several characteristics of the central nervous system (CNS), particularly those that involve learning, memory and mood related responses^10, 13^. POP is characterized by an overall cylindrical shape that is constituted by two main domains^14^ (Fig. 1). The peptidase or catalytic domain (residues 1–72 and 428–710) consists of a typical α/β hydrolase fold. A seven-bladed β-propeller domain (residues 73 to 427) is attached to the C-terminal of the catalytic domain. The active site is located in a large cavity at the interface of these two domains and is shaped by a catalytic triad that contains Ser554, Asp641, and His680 residues (Fig. 1A). The active site is further grouped into several subsites. The S1 specificity pocket is formed by the side chains of Phe476, Asn555, Val580, Trp595, Tyr599, and Val644, which form a hydrophobic environment for the easy fit of the substrate proline or the aromatic rings of the inhibitors. The portion of the S2 pocket that contains the guanidinium side-chain of Arg643 is comparatively less specific. The S3 pocket is formed by the side chains of many nonpolar residues, including Phe173, Met235, Cys255, Ile591, and Ala594, which creates a relatively large hydrophobic environment.

Considerable alterations in POP activity have been reported in many neurological conditions, such as Alzheimer’s disease, Parkinson’s disease, Huntington’s disease, mania, clinical depression, dementia, and autism; however, its precise physiological role in pathogenesis remains unclear^15^. Recent studies have suggested that POP directly interacts with alpha-synuclein (aSyn) and increases its dimerization, which is one of the key factors in the pathology of Parkinson’s disease (PD)^16, 17^. Macroautophagy is a cellular mechanism that is crucial for oligomeric aSyn clearance in synucleinopathies. The inhibition of POP induces the expression of beclin 1, which is a positive regulator of autophagy that promotes the removal of aSyn protein aggregates^18^. Therefore, POP has been proposed to be a potential therapeutic target in the treatment of synucleinopathies. Additionally, POP is involved in the cell cycle progression^19^ and is elevated in many cancers with a possible regulatory role in angiogenesis promotion^20, 21^. POP has also been considered as a potential target for the treatment of Chagas disease, leishmaniasis, and African trypanosomiasis^22, 23^.

There have been continuous efforts to inhibit POP as a treatment for several neurodegenerative disorders^24, 25^. N-benzyloxycarbonyl prolyl-prolinal (ZPP)^26^, which is a substrate-like peptidomimetic compound, was the first discovered POP inhibitor. Thereafter, several small molecules that are capable of inhibiting POP have been reported, and these molecules have anti-amnesic, cognition-enhancing and neuroprotective properties^24–28^. However, the majority of the repertoire of synthetic POP inhibitors is derived from a systematical modification of ZPP. Most of the reported POP inhibitors are substrate-like short pseudopeptides that have a small potential of crossing the blood-brain barrier (BBB), which is a very unfavourable pharmacokinetics characteristic in drug design for CNS diseases. Hence, it is evident that even though there are many patents registered for POP inhibitors, only two compounds (Z-321and S-17092) have reached clinical trials in humans^27^. Therefore, there is a need to identify potent POP inhibitors that have better pharmacodynamics and pharmacokinetics properties in addition to having a non-peptidic nature.

Several POP inhibitors have been patented, most of which consist mainly of substrate-like peptidomimetics; however, certain alkaloids and heteroaryl ketone inhibitors have also been reported^27^. Very few non-peptidic POP inhibitors have been reported, including natural products, such as berberine and baicalein. However, their poor selectivity and low activity limit their use as potent POP inhibitors. A comprehensive review of POP inhibitors has been published by Lawandi *et al*.^24^. The information regarding the reported inhibitors indicated that the existing peptidomimetic inhibitors form complementary interactions at the active site of POP, which is represented by the pharmacophore shown in Fig. 1B. The P1′ portion of the inhibitor is often formed by reactive groups, such as CHO, C(O)CH_2_OH, and CN, which establish a covalent bond with the catalytic Ser554 in the S1′ pocket. The P1 usually comprises a hydrophobic group to occupy the S1′ pocket. The P2 portion is relatively less specific compared to P1 and frequently contains Arg or Pro residues. The P3 part is hydrophobic, has no size restrictions and may contain bulky aliphatic and aromatic groups. Two carbonyl groups of peptidic inhibitors are involved in hydrogen bond interactions with the Trp595 and Arg643 residues. To develop more potent and selective POP inhibitors, a better understanding of the above-mentioned key inhibitor sites is essential for medicinal chemists^24, 27^.

In the present study, a strategy amalgamating pharmacophore-based virtual screening, molecular docking, *in vitro* assays, and molecular dynamics (MD) simulations were employed to identify new scaffolds for the design of POP inhibitors with a non-peptidic nature. To enhance the reliability and efficiency of the computer aided drug-design approaches, recent advances in pharmacophore modelling strategies recommend the use of combined information from both the ligands and the protein^29^. Hence, the available POP structural information and the pharmacophores of its known inhibitors were exploited for the generation of structure-based and ligand-based pharmacophore models, respectively. A drug-like database was prepared and used for the pharmacophore-based virtual screening to obtain potential leads for POP inhibition. Compounds that were capable of penetrating the BBB were considered of utmost importance and selected. Further screening of the compounds were performed by molecular docking and the representative hits were tested for *in vitro* POP inhibition and the levels of aSyn expression in over-expressing SH-SY5Y human neuroblastoma cells were analysed. The binding mode analysis of the final hit compounds was carried out by MD simulations.

## Results and Discussion

**Figure 2:**
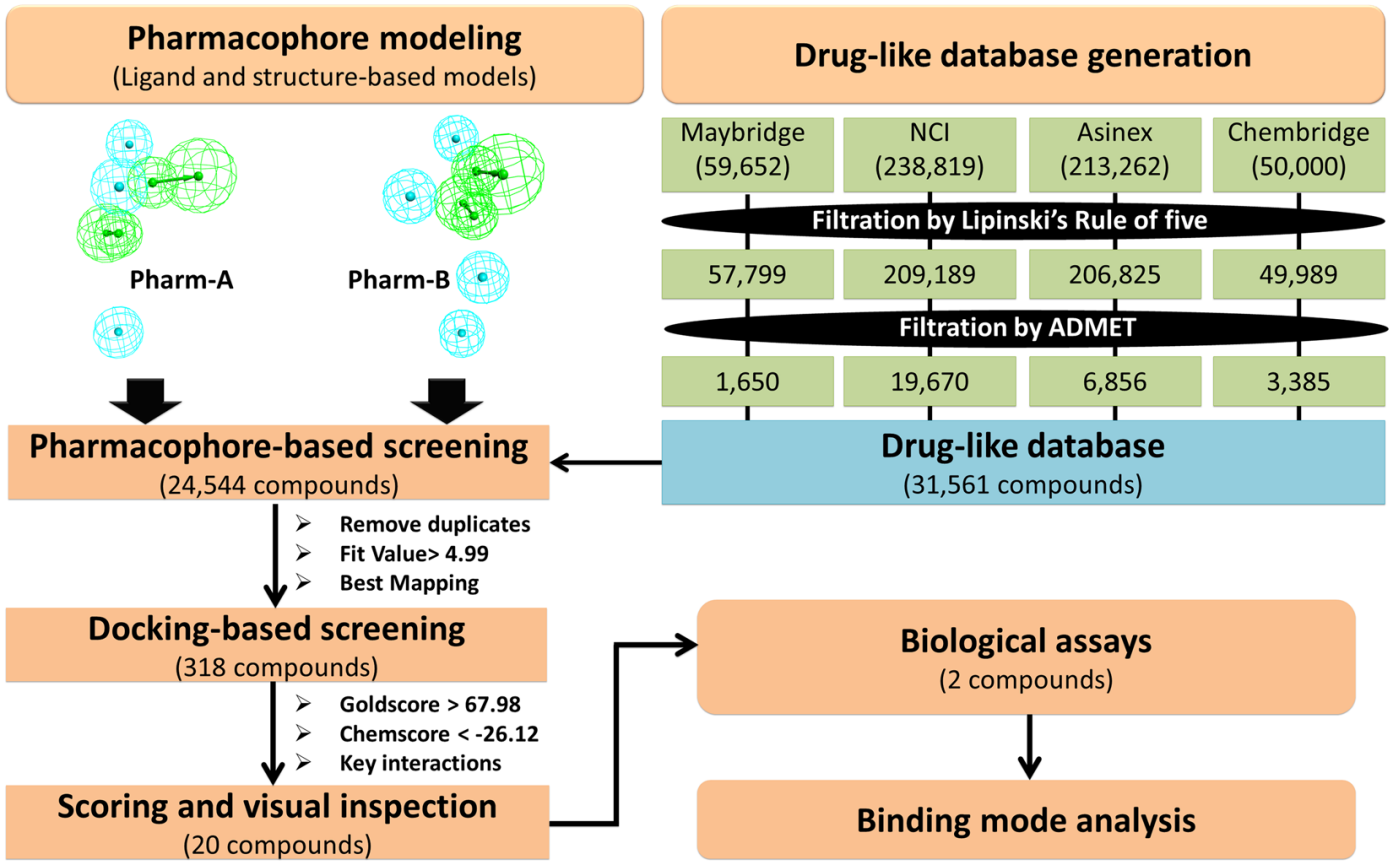
Schematic workflow of the virtual screening for identifying the prolyl oligopeptidase inhibitors.

Integrated computational approaches, including pharmacophore-based virtual screening, molecular docking, *in vitro* biological assays, and MD simulations were employed to identify potential leads for POP inhibition. The strategy used in the present study is presented as a workflow in Fig. 2.

### Common-feature pharmacophore generation

**Figure 3:**
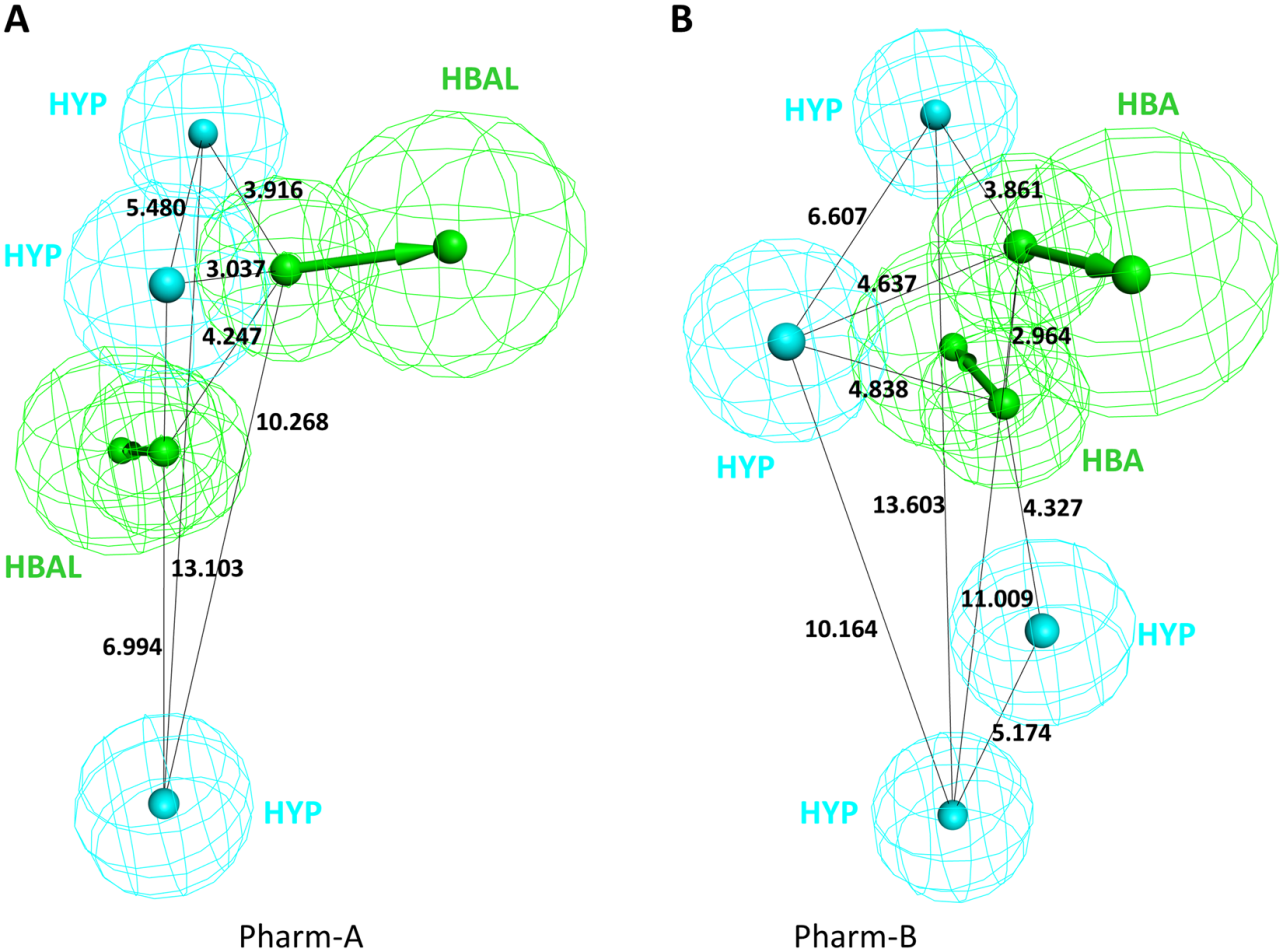
Pharmacophore models of POP generated with distance constraints. (**A**) Common feature pharmacophore, Pharm-A; (**B**) Structure-based pharmacophore, Pharm-B.

Ligand-based strategies use the innate chemical features of a set of compounds to represent the critical interactions between the ligand and a specific target macromolecule. Eight well-known POP inhibitors were used as the training set for ligand-based pharmacophore generation (Supplementary Figure S1). All compounds represented sufficient structural diversities, which was required for the generation of the pharmacophore models. The compounds Z-321 (Inh 1) and S-17092 (Inh 2) were given more weighting during the pharmacophore generation and were used as reference inhibitors in our study. In total, 10 pharmacophore models were generated, and their statistical values were obtained (Table 1). All hypotheses contained the combination of at least two or more pharmacophore features, such as a hydrogen bond acceptor lipid (HBAL), hydrogen bond acceptor projection (HBAP), ring aromatic (RA), and hydrophobic (HYP). The best pharmacophore model was selected based on its rank and the alignment of the ligands to the pharmacophore model at the active site of POP. The rank of the obtained models was in the range of 86.22 to 88.67 with a maximum fit of 5. The 10 models differed in their chemical features and spatial arrangements. Therefore, the generated pharmacophore models were overlaid onto the active site of POP, which revealed that the pharmacophore model 6 (Pharm-A) matched well with the important complementary residues (Supplementary Figure S2). The rank of Pharm-A was observed to be 86.68 (Table 1). Pharm-A contained 2 HBAL, and 3 HYP features with fit values of 4.99 and 2.84 for compounds Z-321 and S-17092, respectively (Fig. 3A, Supplementary Table S1). Thus, the fit value of 4.99 was used as the cut-off in the screening of the chemical databases for the validation and virtual screening. Pharm-A was further validated in terms of its reliability in screening potential POP inhibitor candidates.

**Table 1:** Statistical parameters and results of the common-feature pharmacophore generation.

No.	Features^a^	Rank	Direct Hit	Partial Hit	Max Fit
1	3 HYP, 2 HBAP	88.67	11111111	00000000	5
2	3 HYP, 2 HBAP	88.67	11111111	00000000	5
3	1 RA, 2 HYP, 1HBAL, 1 HBAP	87.58	11111111	00000000	5
4	3 HYP, 1 HBAL, 1 HBAP	87.56	11111111	00000000	5
5	1 RA, 2 HYP, 2 HBAP	87.12	11111111	00000000	5
6	3 HYP, 2 HBAL	86.68	11111111	00000000	5
7	1 RA, 2 HYP, 2 HBAL	86.62	11111111	00000000	5
8	1 RA, 2 HYP, 2 HBAL	86.41	11111111	00000000	5
9	1 RA, 2 HYP, 2 HBAL	86.33	11111111	00000000	5
10	1 RA, 2 HYP, 2 HBAL	86.22	11111111	00000000	5

^a^HBAL, hydrogen bond acceptor lipid; HBAP, hydrogen bond acceptor projection; HYP, hydrophobic; RA, ring aromatic.

### Structure-based pharmacophore generation

**Figure 4:**
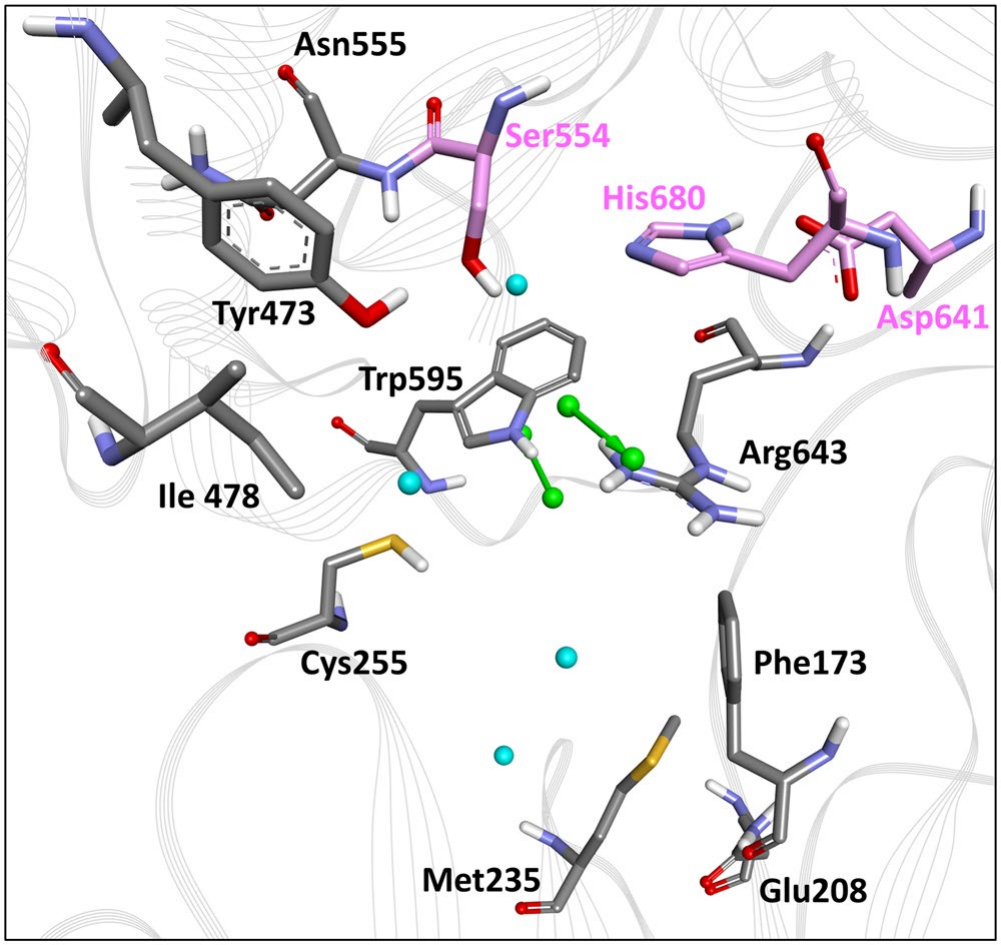
Clustered structure-based pharmacophore model in the active site of POP. Hydrogen bond acceptors and hydrophobic features are shown in green and cyan colours, respectively. Active site residues are shown as stick models in grey, while the catalytic residues are shown in pink. The protein is represented by the grey line ribbons. The pharmacophore features were represented without location spheres for clarity.

The structure of the human POP (PDB ID: 3DDU) with a bound inhibitor was utilized to generate pharmacophores that are complimentary to its active site. A well-defined active site for substrate/inhibitor binding contains high conserved catalytic active site residues, i.e., Ser554, Asp641, and His680^30^. POP has a highly similar sequence and structure^24^. The conserved residues at the active site of POP, which was obtained from different sources, bind different inhibitors in a similar fashion (Supplementary Figure S3). Based on this analysis, the important complementary pharmacophoric features that were generated at the active site of human POP were further sorted. The final structure-based pharmacophore model (Pharm-B) contained 2 HBA and 4 HYP features (Fig. 3B). The two HBA features were complementary to Trp595 and Arg643. Two of the HYP features were lined against the S1 pocket residues, such as Ile478, Asn555, Val580, and Trp595. The other two HYP features were plotted near residues Phe173 and Met235 in the S3 pocket (Fig. 4). The compounds that mapped on to most of these identified features can have the potential to inhibit POP with a high efficacy. For additional validation, the eight training set compounds that were used to generate the common-feature pharmacophore model were mapped with Pharm-B. The fit values of these compounds were calculated and compared. Z-321 had the highest fit value of 4.65, while S-17092 represented a fit value of 4.01 (Supplementary Table S1). Both Z-321 and S-17092 mapped with the two HBA and three HYP features of Pharm-B (Supplementary Figure S4). These mapping results indicated that Pharm-B can map potent compounds from chemical databases by virtual screening methodologies.

### Comparison of the generated common-feature and structure-based pharmacophore models

A comparison of the Pharm-A and Pharm-B pharmacophore features was performed. Pharm-A consisted of five chemical features, including two HBAL and three HYP features, while Pharm-B consisted of six features, including containing 2 HBA and 4 HYP features. The 2 HBAL and 2 HBA features of the Pharm-A and Pharm-B models can receive hydrogen atoms from the Trp595 and Arg643 residues. The distance between the respective acceptor features was 4.247 Å and 2.964 Å in Pharm-A and Pharm-B, respectively (Fig. 3). Pharm-A had 3 HYP features, whereas Pharm-B contained 4 HYP features. Both models contained complementary HYP features for the S1 and S3 pockets. The distance between the S1 and S3 HYP features was 13.103 Å and 13.603 Å, respectively. An additional HYP feature in Pharm-B was lined against the Phe173 residue, which may promote π-π interactions with the inhibitors as reviewed by Lawandi *et al*.^24^.

### Güner-Henry (GH) scoring method for the validation of the pharmacophore models

The GH scoring method was used to determine how well our pharmacophore models can discriminate active POP inhibitors from inactive POP inhibitors. An internal database containing 57 active POP inhibitors (Supplementary Table S2) and 2000 decoy compounds was prepared (Table 2). Both the Pharm-A and Pharm-B pharmacophore models were employed to map the active compounds in the database. All compounds were allowed to map on to all features of Pharm-A, while one of the six features was allowed to be excluded in Pharm-B. Pharm-A retrieved 87.72% of the active compounds and 17 inactive compounds (false positives) and predicted that 7 active compounds were inactive (false negatives). However, Pharm-B mapped 92.98% of the active compounds in addition to predicting 11 false positives and 4 false negative compounds. The enrichment factor reflects the specificity and selectivity of the model, as it specifies the concentration (or enrichment) of active compounds in the model relative to the screening of the database containing decoys. Pharm-B exhibited a higher enrichment factor value (29.88) than Pharm-A (26.93). Generally, the goodness of fit (GH) scores ranged from 0, which indicates a null model, to 1, which indicates an ideal model^31^. Therefore, a model with a GH score that is greater than 0.6 and closer to 1 is expected to be more reliable in the screening of large chemical databases^32^. Both Pharm-A and Pharm-B exhibited good GH scores of 0.84 and 0.85, respectively. Various researchers have previously reported using models with a GH score range from 0.67 to 0.86^33–35^. These results demonstrate the suitability of Pharm-A and Pharm-B for database screening to map potential leads that are capable of POP inhibition.

**Table 2:** Statistical parameters obtained by the Güner-Henry (GH) scoring method used for validation of Pharmacophore models.

S.No.	Parameter	Pharm-A	Pharm-B
1	Total number of molecules in database (D)	2057	2057
2	Total number of actives in database (A)	57	57
3	Total number of hit molecules from the database (Ht)	67	64
4	Total number of active molecules in hit list (Ha)	50	53
5	% Yield of actives [(Ha/Ht) × 100]	74.63	82.81
6	% Ratio of actives [(Ha/A) × 100]	87.72	92.98
7	Enrichment factor (EF) [(Ha X D)/(Ht X A)]	26.93	29.88
8	False negatives [A-Ha]	7	4
9	False Positives [Ht – Ha]	17	11
10	Goodness of fit ((Ha/4HtA)(3A + Ht))X ((1-((Ht-Ha)/(D-A))	0.84	0.85

### Generation of drug-like database and pharmacophore-based virtual screening

Although POP is broadly distributed in the human body, it is found in high concentrations in the brain. Therefore, the potential inhibitors must cross the blood-brain barrier to be effective against POP-related CNS disorders. Therefore, compounds that are capable of crossing the BBB in addition to having essential drug-like characteristics with minimum toxicity should be considered for virtual screening. Hence, a drug-like database was established, which comprised compounds that satisfied Lipinski’s rule of five and ADMET criteria. ADMET properties, such as a compound’s ability to penetrate BBB, solubility, human intestinal adsorption (HIA), hepatotoxicity and Cytochrome P450 2D6 (CYP2D6) inhibition, were measured. After its oral administration, the potential drug compound should be absorbed (at least 90%) by the human intestine from the bloodstream^36, 37^. Thus, the absorption level was set to 0, which represents good absorption properties of compounds. Compounds that are capable of penetrating the BBB with a high strength were sorted by setting the value of the BBB to 2. The aqueous solubility of each compound in water at 25 °C was calculated. A solubility level of 3 represents a good solubility property and compounds reaching this level were selected for further screening. Compounds with a low hepatotoxicity and CYP2D6 inhibition were selected for further studies. Finally, the remaining compounds were compiled to form a drug-like database that was used in the virtual screening process (Fig. 2).

Pharmacophore model-based methods have been extensively used for the virtual screening of chemical databases to reduce the costs of high throughput screening in drug discovery and development processes. Moreover, database screening is advantageous over *de novo* design methods owing to the easy identification of potential leads that are suitable for further optimization and synthesis^38^. Pharm-A and Pharm-B mapped 19,785 and 5,759 compounds, respectively. A fit value of 4.99 was used to further refine the compounds. Duplicate compounds were removed, and the remaining compounds were manually observed for best mappings onto the pharmacophore models. Consequently, 318 compounds were obtained and subjected to the molecular docking studies to assess their binding orientation at the active site of POP.

### Docking-based screening

The suitability of the genetic algorithm in our study was assessed before the molecular docking calculations. The bound co-crystal ligand from the POP structure (PDB ID: 3DDU) was docked into the X-ray structure (Supplementary Figure S5). The docking pose was generated with a very low RMSD value of 0.09 Å, which indicated the reliability of GOLD in the present study. Subsequently, 318 compounds, along with reference compounds Inh 1 and Inh 2, were docked into the active site of POP using the same parameters. The best pose for each ligand was selected based on a high Goldscore, a low Chemscore and important molecular interactions between the ligand and the active site of POP. The Goldscore for Inh 1 and Inh 2 was 67.98 and 56.89, respectively. The Chemscore binding energy for Inh 1 and Inh 2 was −26.12 and −25.78 kJ/mol, respectively. Therefore, a Goldscore cut-off value of 67.98 and a Chemscore cut-off value of −26.12 were used to further screen the binding poses. Consequently, 20 compounds that showed better scores were selected (Supplementary Table 3). Moreover, the compounds were assessed manually for their interactions with the active site residues of POP. As reviewed above, the active site of POP has been divided into several subsites. The hydrophobic residues, such as Trp595, Phe476, Val644, Val580, and Tyr599, form the so-called S1 pocket, which allows the proline ring of its substrate to fit tightly and stack with the indole ring of Trp595. Inhibitors that contain a hydrophobic group that is complementary to an S3 pocket lined with nonpolar residues, such as Phe173, Met235, Cys255, Ile591, and Ala594, characterize tight binding to POP. Moreover, the hydrogen bond formation with Arg643 and the electrostatic interactions with catalytic residues, such as Ser554, Asp641, and His680, have been considered critical for POP inhibition^30^. Only 4 compounds represented all important interactions that are required for effective POP inhibition. The Goldscores and the Chemscores values of the top scoring four compounds were higher as compared to reference inhibitors (Supplementary Table 3). The Goldscores of compounds 1 to 4 were 74.34, 69.93, 69.73, and 69.64, whereas, the Chemscores were −34.68, −34.18, −32.89, and −33.69, respectively. We purchased two out of the top scoring molecules from their respective databases and tested them for *in vitro* assays against the POP activity and SH-SY5Y human neuroblastoma cells.

### Hit compounds inhibit POP enzyme activity *in vitro*

**Figure 5:**
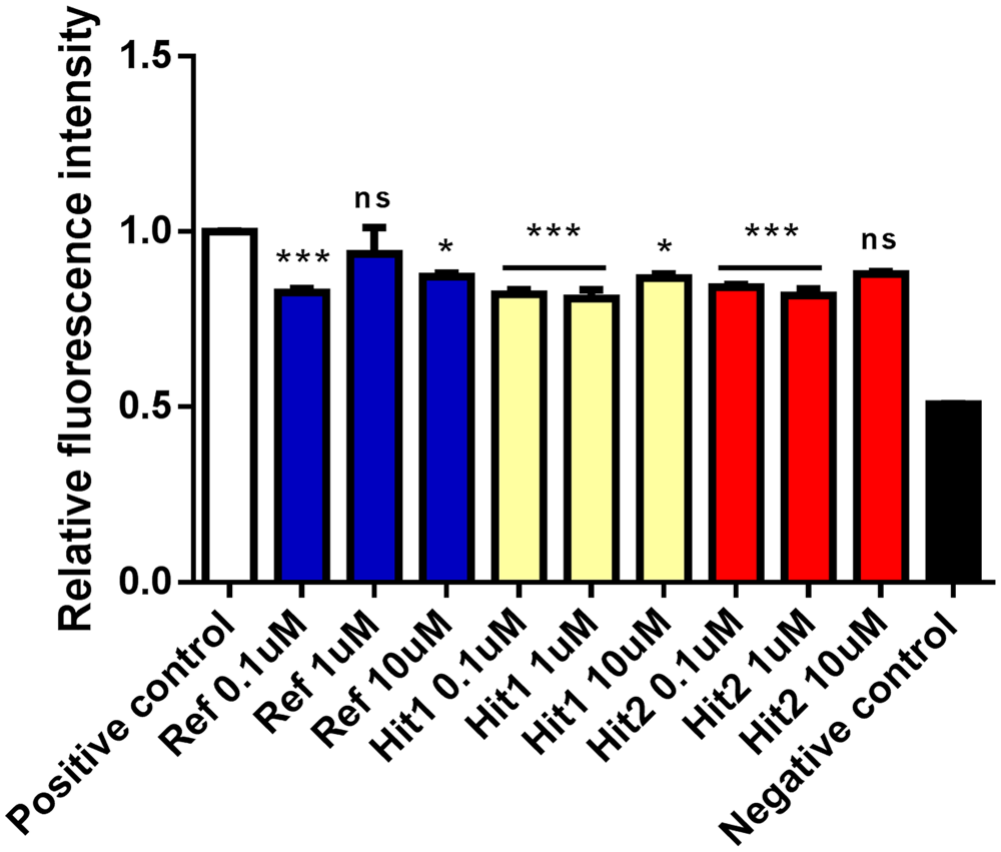
The effect of hit compounds on enzyme activity determined by *in vitro* POP assay. All samples are divided into the following 11 groups: positive control, negative control, Reference inhibitor (Ref), Hit 1 and Hit 2 compounds at three different doses (0.1, 1 and 10 µM). The data are shown as the mean ± SEM of triplicates from three independent *in vitro* experiments. *p < 0.05, ***p < 0.001 and ns *p* > 0.05 compared to positive control.

We speculated that the hit compounds that were identified by our computational approaches are potential candidates for POP inhibition by blocking its active site. To verify our hypothesis, we analyzed the effect of the Hit 1 and Hit 2 compounds by POP assay *in vitro*. POP enzyme activity was measured in order to verify inhibition of POP enzyme against reference inhibitor, Hit 1 and Hit 2 compounds (Fig. 5). The *in vitro* POP assay results of the hit compounds were compared with results obtained with the well-known POP inhibitor S17092 (training set compound, Inh 2), which was used as a reference compound. The Hit 1 and Hit 2 compounds significantly decreased the activity of POP at the lower concentrations similarly to the reference inhibitor [Hit 1 (0.1, 1 µM ***p < 0.001, 10 µM *p < 0.05), Hit 2 (0.1, 1 µM ***p < 0.001, 10 µM ns > 0.05)]. The reference inhibitor was active against POP at a low concentration of 0.1 µM. In comparison, the Hit 1 and Hit 2 compounds were capable of inhibiting the POP activity at a wide range of concentration (0.1 to 10 µM). All compounds were active against POP at lower concentration (0.1 µM) while showing slight variations at higher concentrations. These results suggest that the Hit 1 and Hit 2 compounds were able to inhibit the activity of the POP enzyme at low concentrations.

### Hit compounds reduce the expression level of α-synuclein *in vitro*

**Figure 6:**
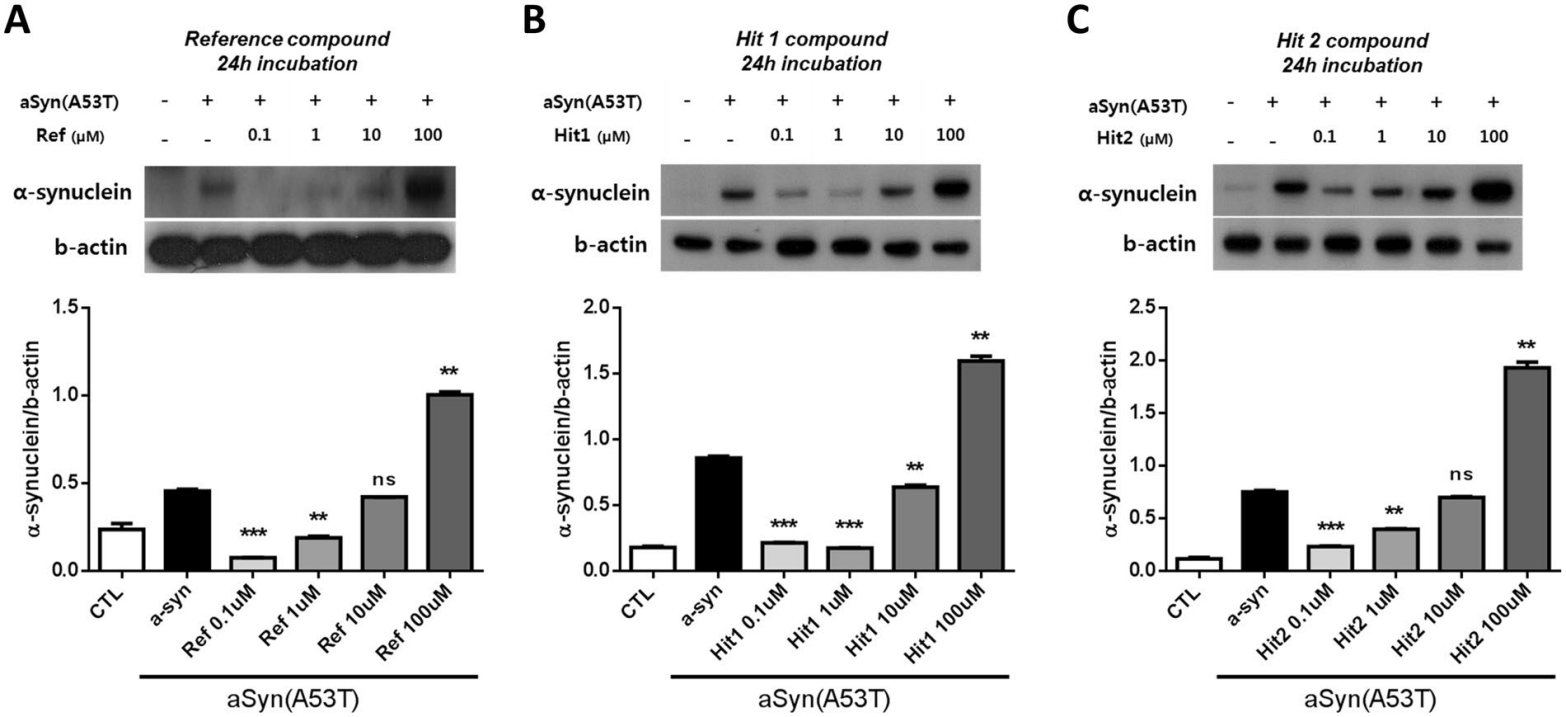
The expression of α-synuclein in SH-SY5Y human neuroblastoma cells treated with the hit compounds. All samples were divided into the following six groups: control SH-SY5Y cells, overexpressing SH-SY5Y cells transfected with EGFP-alphasynuclein-A53T plasmid (α-syn), overexpressing SH-SY5Y cells treated with (**A**) Reference inhibitor, (**B**) Hit 1, and (**C**) Hit 2 compounds at four different doses (0.1, 1, 10, and 100 µM for 24 h). Western blots were stained with anti-α-synuclein, and anti-b-actin was used as a loading control. The data are shown as the mean ± SEM of triplicates from three independent *in vitro* experiments. The histogram represents the optical density-based quantification of α-synuclein/b-actin; **p < 0.01, ***p < 0.001 and ns *p* > 0.05.

Parkinson’s disease is a progressive and neurodegenerative disorder that is characterized by a loss of terminals in the striatum and is accompanied by the accumulation of α-synuclein (aSyn) aggregates known as Lewy bodies. POP accelerates the aggregation of aSyn, thus facilitating the development of Parkinson’s disease and other synucleinopathies^16^. Previous reports indicated that POP inhibitors reduce the aSyn aggregation and promote the clearance of the aSyn aggregates in aSyn-overexpressing cells and *in vivo* models^17, 39, 40^. Therefore, we analysed the effect of the Hit 1 and Hit 2 compounds on the expression and aggregation of aSyn in over-expressing SH-SY5Y human neuroblastoma cells *in vitro*. The preliminary results of the MTT assays showed that the Ref and hit compounds showed no detectable toxicity at a range of 0.1 µM to 10 µM (Supplementary Figure S6). However, a decrease in the cell viability was observed in cells that were treated with the Ref and hit compounds at 100 µM. Then, the effect of the Hit 1 and Hit 2 compounds on the expression of aSyn in the over-expressing SH-SY5Y cells was examined using a concentration range of 0.1 µM to 100 µM (Fig. 6). The aSyn overexpression was clearly evident in the transfected cells compared to the expression of aSyn in the control cells. The Hit 1 and Hit 2 compounds significantly decreased the expression level of α-synuclein at the lower concentrations similarly to the reference inhibitor [Hit 1 (0.1, 1 µM ***p < 0.001, 10 µM **p < 0.01), Hit 2 (0.1 ***p < 0.001, 1 µM **p < 0.01, 10 µM ns > 0.05)] (Fig. 6A,B and C). Compared to the reference inhibitor, Hit 1 is capable of decreasing α-synuclein expression at a wide range of concentrations (0.1 to 10 µM). Interestingly, a significant overexpression of α-synuclein was observed in all treatments at the higher concentration of 100 µM. In conclusion, the Hit 1 and Hit 2 compounds were able to attenuate the expression level of α-synuclein at low concentrations in the SH-SY5Y cells.

### Binding mode analysis of the hits by molecular dynamics simulations

**Figure 7:**
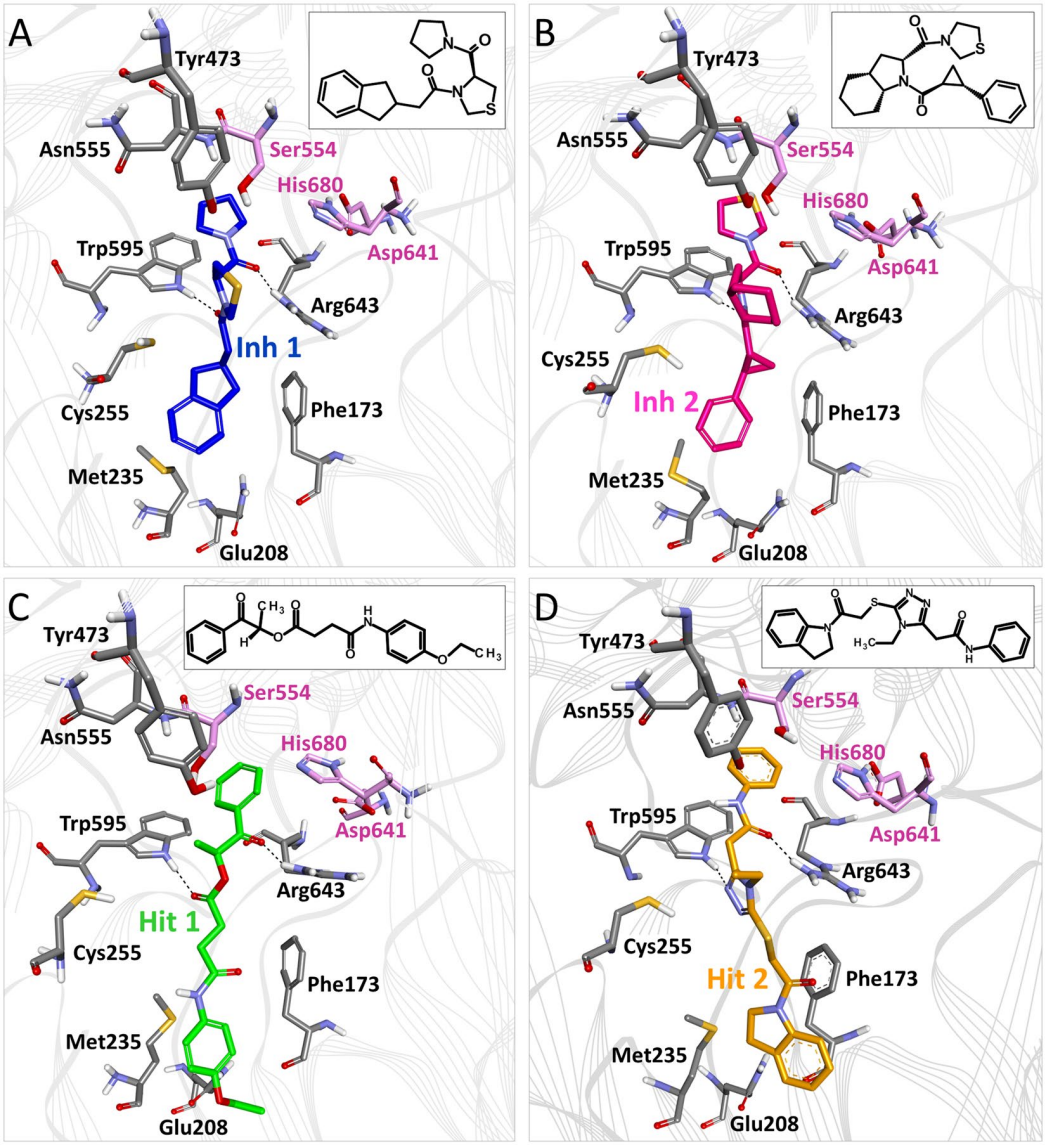
A pictorial representation of binding modes of the reference inhibitors and hit compounds. (**A**) Inh 1 (**B**) Inh 2 (**C**) Hit 1 (**D**) Hit 2. The 2D structures of all compounds are shown as insets. The active site residues are represented by a grey stick model, while the catalytic residues are shown in pink. The protein is shown in line ribbons. Hydrogen bonds are displayed as dotted lines. Only polar hydrogen atoms are shown for clarity.

MD simulations approaches have successfully been employed to study complex molecular behaviours at the atomic level by comparing experimental results and theory, molecular motions and function, modelling enzyme mechanisms and complex biomolecular assemblies^41^. Here, the purpose of the MD simulations was to assess the binding stability of the final hit-POP complexes. Each system contained a POP protein in a complex with its ligand in a cubic box containing TIP3P water molecules and counter ions (Supplementary Table S4). The 20 ns MD simulations were carried out for each system. The root mean square deviation (RMSD) values of the protein backbone atoms and the potential energies of the systems were calculated to assess the overall protein stability throughout the simulation (Supplementary Figure S7). The systems were well converged and the average RMSD values for the protein backbone atoms were found to be 0.09 nm, 0.10 nm, 0.11 nm, and 0.11 nm for Inh 1, Inh 2, Hit 1, and Hit 2, respectively. In addition, the potential energies for the systems remained constant relative to the normal behaviour of the protein throughout the 20-ns simulation. Compounds Z-321 (Inh 1) and S-17092 (Inh 2) from the training set were used as the reference for comparison with the hit compounds. The trajectories from the last 5 ns of the total simulation time were obtained to elucidate the reliable binding modes of the hit compounds with POP. Upon superimposition, it was recognized that all hit compounds and reference inhibitors bind with POP at the same substrate/inhibitor binding cavity (Supplementary Figure S8). The pyrrolidine ring of Inh 1 fitted well into the specificity-determining S1 pocket of the POP active site, and two carbonyl groups formed hydrogen bond interactions with important amino acids such as Trp595 and Arg643 (Fig. 7A, Table 3). Similar binding was observed in the case of Inh 2, in which the thiazolidine ring occupied the S1 pocket, and two carbonyl groups formed hydrogen bonds with Trp595 and Arg643 (Fig. 7B). Both inhibitors exhibited π-π interactions with the aromatic side chain of Trp595 in the S1 hydrophobic pocket and contained bulky hydrophobic rings that occupied the S3 pocket. Moreover, the reference inhibitors represented electrostatic interactions with Ser554, Trp595, and Arg643. In addition to the specificity-determining S1 pocket residues Phe476, Val580, and Val644, both Inh 1 and Inh 2 showed hydrophobic interactions with S3 pocket residues, including Met235, Cys255, Ile478, Ile591, and Ala594 (Table 3). Hit 1 formed strong hydrogen bonds with residues Trp595 and Arg643, and represented electrostatic interactions with Gln208, Gly237, Cys255, Trp595, and Arg643 (Fig. 7C). Hit 1 also showed hydrophobic interactions with the Phe476, Val580, and Val644 residues in S1 as well as the Phe173, Met235, Cys255, Ile478, Ile591, and Ala594 residues in the S3 pocket. Hit 2 formed hydrogen bonds with Trp595 and Arg643 (Fig. 7D). The binding mode of Hit 2 revealed electrostatic interactions with the catalytic residue Ser554 in addition to its interactions with Cys255, Trp595, and Arg643. Furthermore, the binding of Hit 2 to POP was confirmed by hydrophobic interactions with residues in the S1 (Phe173, Met235, Cys255, Ile591, and Ala594) and S3 (Phe476, Ile478, Val580, and Val644) pocket. Additionally, cation-π interactions of the triazol ring of Hit 2 with Arg643 were also observed. Moreover, the number of hydrogen bonds between POP and each compound were monitored during the 20 ns simulations (Supplementary Figure S9). Both Inh 1 and Inh 2 maintained an average number of 1.733 and 1.883 hydrogen bonds throughout the simulation. The hit compounds showed more than the average number of hydrogen bonds during the simulations. The average numbers of hydrogen bonds between the hit compounds and POP were 1.911 and 1.905 for Hit 1 and Hit 2, respectively. The hydrogen bond formation between the inhibitors and the Arg643 residue of the active site is one of the prerequisites for POP inhibition^42^. Therefore, the hydrogen bonds between Arg643 and the respective ligands in this study were monitored. The results showed that hit compounds, along with the reference inhibitors, maintained a hydrogen bond with Arg643 during the 20 ns simulations, indicating the potential of the hit compounds to inhibit POP. Moreover, the different aromatic rings of the hit compounds showed a π-π stacking interaction with Trp595, which was similar to those observed in most of the known inhibitors, including the reference compounds. The hit compounds consistently showed van der Waals interactions with residues, such as Phe173, Met235, Arg252, Phe476, Ile478, Ser554, Ile591, Ala594, Val644, and His680. Details regarding the various interacting residues are documented in Table 3.

**Table 3:** Various molecular interactions identified between the compounds and the POP protein.

Name	Hydrogen bond interaction				π – π Interaction	Electrostatic Interaction	Hydrophobic interaction
	Ligand atom	Amino acid	Amino acid atom	Length (Å)			
Inh 1	O2	Trp595	HE1	1.849	Trp595	Ser554, Trp595, Arg643	Met235, Cys255, Phe476, Ile478, Val580, Ile591, Ala594, Val644
	O1	Arg643	HH11	1.599			
Inh 2	O2	Trp595	HE1	1.733	Trp595	Ser554, Trp595, Arg643	Met235, Cys255, Phe476, Ile478, Val580, Ile591, Ala594, Val644
	O1	Arg643	HH11	1.826			
Hit 1	O3	Trp595	HE1	2.014	Trp595	Gln208, Gly237, Cys255, Trp595, Arg643	Phe173, Met235, Cys255, Phe476, Ile478, Val580, Ile591, Ala594, Val644
	O1	Arg643	HH11	1.631			
Hit 2	N5	Trp595	HE1	2.190	Trp595, His680	Cys255, Ser554, Trp595, Arg643	Phe173, Met235, Cys255, Phe476, Ile478, Val580, Ile591, Ala594, Val644
	O1	Arg643	HH11	3.042			

Subsequently, it was revealed that the hit compounds bind to the POP active site by various molecular interactions, such as hydrogen bonds, electrostatic interactions, hydrophobic interactions, and van der Waals interactions. Compared to the reference compounds, the hit compounds showed consistent hydrogen bond interactions and greater electrostatic interactions with important amino acids. Moreover, the hit compounds interacted with the specificity-determining S1 pocket residues along with the hydrophobic S3 pocket, which demonstrates the potential of these compounds to act as potent POP inhibitors. The 2D structures of the remaining compounds from the virtual screening are represented in Supplementary Figure S10. A similarity search was carried out for the hit compounds using the *PubChem Structure* ^43^ and *ChemSpider* ^44^ online server tools. The search results confirmed that our hit compounds have not been previously experimentally tested for their POP inhibiting potential.

## Conclusion

POP is a widely studied therapeutic target, and experimental data have demonstrated that POP inhibitors have anti-amnesic, cognition-enhancing and neuroprotective properties. Most inhibitor design strategies previously centered on Z prolyl-prolinal based derivatives. Although several peptidomimetic inhibitors have been developed, only two compounds, i.e., Z-321 and S-17092, have reached clinical trials. The most important reported reason for the failure of most inhibitors during the clinical trials was their inability to cross the blood-brain barrier to penetrate and distribute inside neurons. To improve the pharmacological properties, the development of inhibitors with a lesser peptidic nature could be a promising choice. Hence, pharmacophore-based virtual screening, molecular docking, and molecular dynamics (MD) simulation techniques were employed to identify potent chemical scaffolds that are capable of POP inhibition in addition to having good pharmacological properties and a non-peptidic nature. Subsequently, a ligand-based pharmacophore model, Pharm-A, and a structure-based pharmacophore model, Pharm-B, were generated. Pharm-A comprises two HBAL and three HYP features, whereas Pharm-B comprises two HBA and four HYP features. The hypotheses were validated by the Güner-Henry (GH) scoring method. Both models represented good GH scores as Pharm-A and Pharm-B mapped 87.72% and 92.98% of the active compounds, respectively. A drug-like database was prepared, which contained compounds with good pharmacological properties especially that have the ability to penetrate the blood-brain barrier. The compounds in this database satisfied the criteria of Lipinski’s rule of five and ADMET properties. Pharm-A and Pharm-B mapped 19,785 and 5,564 compounds from the drug-like database screening, respectively. A molecular docking analysis was carried out to assess the capability of these compounds to bind with important amino acids in the POP active site and monitor their binding conformations. The top compounds having a high Goldscore, low Chemscore, and important molecular interactions with POP were retained. As a result of docking based screening, 20 compounds were obtained and out of top scoring compounds, two compounds were chosen for *in vitro* POP assays. The hit compounds were capable of inhibiting POP activity at a wide range of concentrations from 0.1 to 10 µM. Although the hit compounds showed slight variations at higher concentrations, they were able to inhibit the activity of the POP enzyme at low concentrations *in vitro*. Additional *in vitro* biological evaluation indicated that compounds Hit 1 and Hit 2 significantly reduced the expression of aSyn expression in overexpressing SH-SY5Y cells. The compounds were effective at low concentrations (0.1 µM to 10 µM), which was comparable to the reference inhibitor. The binding modes of the hit compounds derived from the MD simulations indicated that they bind at the same cavity in POP as has been reported previously^14, 30^. Moreover, it was observed that the hit compounds bind to the POP active site by numerous molecular interactions, such as hydrogen bonds, electrostatic interactions, hydrophobic interactions, and van der Waals interactions. The hit compounds formed hydrogen bonds with important amino acids, such as Trp595 and Arg643, and represented a greater extent of electrostatic interactions compared to the electrostatic interactions formed by reference inhibitors. The hit compounds represented high numbers of hydrogen bond formations during the simulations compared to those of the known inhibitors. Moreover, the hit compounds exhibited hydrophobic interactions with the specificity-determining pocket S1 and the hydrophobic pocket S3 residues. Therefore, the Hit 1 and Hit 2 compounds are ideal candidates for POP inhibition by blocking the active site of POP. A similarity search analysis proved that our hit compounds have not been previously tested experimentally for POP inhibition.

Finally, our findings suggest that the hit compounds that were identified through our computational analysis can present potential candidates for the treatment of Parkinson’s disease by inhibiting the active site of POP. Moreover, we believe that the new scaffolds that included our final hits will be helpful for designing novel drugs for the treatment of POP related CNS disorders, such as Alzheimer’s disease (AD), and other α-synucleinopathies, including Parkinson’s disease (PD), dementia with Lewy bodies (DLB), and multiple system atrophy (MSA).

## Methods

### Common feature pharmacophore generation

The chemical features of 8 well-known POP inhibitors were utilized for the ligand-based pharmacophore generation. The model generation was accomplished using the *HipHop* algorithm. *HipHop* is implemented as a *Common Feature Pharmacophore Generation* protocol in Discovery Studio v3.5 (DS). All compounds were assessed for the non-violation of Lipinski’s rule of five; in particular, the molecular weight of the compound must be less than 500 Da. More weighting was given to Z-321 and S-17092 by setting the principal value and *Maximum Omitted Features* to 2 and 0, respectively. The inherent chemical features of the compounds used in the training set were examined by the *Feature Mapping* protocol in DS. Subsequently, hydrogen bond acceptor (HBA), hydrogen bond acceptor lipid (HBAL), hydrogen bond acceptor projection (HBAP), ring aromatic (RA), and hydrophobic (HYP) chemical functions were selected for the pharmacophore generation. The BEST poling algorithm was used to generate a maximum of 255 conformations at a relative energy threshold of 20 kcal/mol. To ensure the identification of closely related chemical features, the minimum inter-feature distance between any close feature points was changed from the default value of 2.97 Å to 1.0 Å. The value of the *Number of Leads that May Miss* and *Feature Misses* remained at the default value of 1. As a result, ten pharmacophore models were produced with various parameters, such as the rank of hypothesis and maximum fit values.

### Structure-based pharmacophore generation

The POP enzyme family in various sources shares a high sequence and structural similarity^24^. However, only one human POP crystal structure (PDB ID: 3DDU) is currently available and it has been utilized for the structure-based pharmacophore generation in the present study^30^. Residues within 6 Å of the bound inhibitor GSK552 were considered for the identification of complimentary pharmacophoric features using the *Interaction Generation* protocol in DS. The important pharmacophoric features that are prerequisites for POP inhibition were retained by removing the redundant features with no catalytic importance using the *Edit and Cluster pharmacophores* tool in DS. Finally, the most important pharmacophoric features that are involved in inhibitor binding were retained in the structure-based pharmacophore model.

### Hypothesis validation

The ability of our pharmacophore models to identify potent inhibitors of POP was assessed by the Güner-Henry method^32, 45^. POP inhibitors whose experimental activity (IC_50_) was determined by the same biological assay method were obtained from the binding database and an internal dataset was prepared^46–51^. Thereafter, 57 active POP inhibitors (IC_50_ less than 100 nmol/L) were sorted and mixed with a decoy set of 2000 compounds to assess the capability of both the ligand-based and structure-based pharmacophore models to identify active compounds. The database screening was performed using the *Ligand Pharmacophore Mapping* protocol in DS. The FAST algorithm was used to generate the conformations. The identified hit molecules from the database (Ht) were selected using a cut-off value of 4.99 and manually monitoring their mapping onto pharmacophore model. Furthermore, several parameters, such as enrichment factor (EF), percent yield of actives (%Y) to assess the selectivity of the model, ratio percentage of actives in the hit list (%A) indicating the coverage of the activity space by the model, false positives, and false negatives, were calculated. Subsequently, other statistical parameters, such as enrichment factor (EF) and goodness of fit score (GH), were calculated by the following formulae:

1 $${\rm{EF}}=({\rm{Ha}}\times {\rm{D}})/({\rm{Ht}}\times {\rm{A}})$$

2 $${\rm{GH}}=({\rm{Ha}}({\rm{3A}}+{\rm{Ht}}))/(4{\rm{HtA}}))\times (1\,-\,({\rm{Ht}}\,-\,{\rm{Ha}})/({\rm{D}}\,-\,{\rm{A}})$$

where Ha = number of active compounds in the hit list, Ht = number of hits retrieved, D = total number of molecules in the database, and A = number of active molecules present in the database.

### Generation of the drug-like database and virtual screening

Owing to the poor pharmacokinetic properties and toxic nature of compounds, many drug candidates fail to perform well in pre-clinical trials in the drug discovery process. Thus, compounds that fail to satisfy the drug-like properties early on should be ruled out. Compounds from four chemical databases, i.e., Maybridge, Chembridge, Asinex, and NCI, were selected for the drug-like database generation. First, the physicochemical properties of the compounds were assessed by filtering with Lipinski’s rule of five^52^. This rule states that an orally active drug compound is more likely to be membrane permeable and easily absorbed by the human body if it has the following: (1) a molecular weight that is less than 500; (2) a logarithm of the partition coefficient between water and 1-octanol (log*P*, representing compound’s lipophilicity) that is less than 5; (3) a number of hydrogen bond donor groups (usually the sum of the hydroxyl and amine groups) that is less than 5; (4) less than 10 hydrogen bond acceptor groups; and (5) a number of rotatable bonds that is less than 10 (Veber’s rule)^53^. Second, the pharmacokinetic properties of the compounds, such as absorption, distribution, metabolism, excretion, and toxicity, were calculated by the *ADMET Descriptors* protocol in DS.

The generated pharmacophore models (Pharm-A and Pharm-B) were used as 3D queries to search the drug-like database. The *Ligand Pharmacophore Mapping* protocol in DS was used to map the compounds that have potential POP inhibitory chemical characteristics. The conformations were generated by the FAST algorithm with the flexible fitting method. The minimum distance between two features was set to 1.0 Å. A maximum of 255 conformations were generated within the relative energy threshold value of 20 kcal/mol. The most suitable compounds were subsequently selected for the molecular docking study.

### Molecular docking

Docking calculations were performed using Genetic Optimization for Ligand Docking (GOLD v5.2.2)^54^. The crystal structure of the human POP (PDB ID: 3DDU)^30^ with a resolution of 1.56 Å was downloaded from the protein data bank (www.rcsb.org). Before docking, all water molecules were removed, and hydrogen atoms were added to the protein. The POP active site was defined within a radius of 6 Å of the bound inhibitor. The Goldscore and Chemscore were used as the default scoring function and rescoring function, respectively. The Goldscore calculates the overall fitting of the ligand in the binding site by considering various factors, such as the H-bonding energy, van der Waals energy, ligand torsion strain, and metal interaction. The Chemscore measures the total free energy change that occurs on the ligand binding, and it considers the hydrogen bonding, hydrophobic-hydrophobic contact area, metal interaction and ligand flexibility. In total, 10 poses were produced for each ligand.

### POP assay

The *Fluorogenic Prolyl OligoPeptidase* (*POP*) *Assay Kit* was purchased from BPS Bioscience (BPS Bioscience, San Diego, CA, USA). The POP assays were performed according to the manufacturer’s guide. Prior to assay, the fluorogenic DPP substrate 1 in DMSO was diluted with DPP assay buffer to make a 50 µM solution. The POP human recombinant enzyme in DPP assay buffer was diluted to 20 ng/$\mu \ell$ with DPP assay buffer. Simultaneously, the AMC fluorescent standard solution was diluted with DPP assay buffer as 1/2 ratio. A ‘Blank’ negative control containing DPP buffer, and a positive control containing the POP enzyme and DPP substrate 1 were used. The total volumes of the reaction mixtures were maintained at 100 $\mu \ell$ using DPP assay buffer. The reaction mixtures were added to the microtiter black plate in triplicates and incubated at 22 °C for 30 min. The excitation wavelengths ranging from 350–380 nm and detection of emitted light ranging from 440–460 nm were measured on a microplate spectrophotometer on the Glomax Multi Detection System (Promega BioSciences, CA, USA).

### Cell Culture and Transfection

SH-SY5Y cells were purchased from Korea Cell Line Bank (KCLB) and cultured in Dulbecco’s Modified Eagle’s Medium (DMEM, Invitrogen, Carlsbad, CA) containing 10% FBS (Invitrogen, Carlsbad, CA) and 1X anti-biotic/anti-mycotic solution (Invitrogen, Carlsbad, CA) at 37 °C with 5% of CO_2_. The EGFP-alphasynuclein-A53T that was used in the transfection was a gift from David Rubinsztein (Addgene, Cambridge, MA, plasmid #40823). The SH-SY5Y cells were transfected using the EGFP-alphasynuclein-A53T plasmid with Lipofectamine 3000 (Invitrogen, Carlsbad, CA) according to the manufacturer’s instructions when the SH-SY5Y cells were 70% confluent.

### Cell viability assay

The viability of the SH-SY5Y cells was estimated using an MTT (3-(4,5-Dimethlthiazol-2-yl)-2,5-Diphenyltetrazolium Bromide) solution (Life Technologies, Oregon, US). The cells (5 × 10^3^cells/well) were seeded in 96-well plates in an incubator containing 5% CO_2_ at 37 °C for approximately 24 h. Then, the cells were treated with Ref, Hit 1 and Hit 2 (0.1 µM, 1 µM, 10 µM, 100 µM) for another 24 h. Additionally, the MTT solution was added for 3 h at 37 °C. The supernatant medium was pipetted, and dimethyl sulfoxide (DMSO) was added to each well for a 20-min incubation on a shaker to achieve solubilization of the formazan crystals. The absorbance values were measured at 560 nm on a microplate spectrophotometer on the Glomax Multi Detection System (Promega BioSciences, CA, USA), and the cell viability was calculated, which was described as a percentage of the control cells.

### Western Blot Analysis

The whole protein form (6 × 10^6^ cells) was extracted at 4 °C in a RIPA Lysis Buffer with the EDTA protein extraction solution consisting of 1X protease and phosphatase inhibitor cocktails (GenDEPOT, Barker, TX). The Bio-Rad protein assay kit (Bio-Rad Laboratories, Hercules, CA) was used to determine the concentration of the protein samples. The lysates (20 µg protein/lane) were separated by 10–12% SDS-PAGE and then transferred to 0.2 µm PVDF Immobilon membranes (Merck Millipore, Billerica, MA). The membranes were blocked with 5% skim milk (BD Difco, Le Pont-De-Claix, France) or 5% Bovine Serum Albumin (BSA, Amresco, Solon, OH) in TBST (10 mM Tris-Hcl, pH 7.5, 150 mM NaCl and 0.1% Tween-20) for 1 h at RT and incubated overnight at 4 °C with the primary antibody. The membranes were exposed to secondary antibodies conjugated with horseradish peroxidase (HRP) for 2 h at RT, and then cross-reacting proteins were detected by EzWestLumiOne (ATTO, Tokyo, Japan) in a dark room. The primary antibody for α-synuclein was purchased from Cell Signalling Technology (Beverly, MA).

### Molecular dynamics simulations

The molecular dynamics (MD) simulations of POP in a complex with inhibitors and final hit compounds were carried out with CHARMM27 all-atom force field using Groningen machine for chemical simulations (GROMACS 4.5.7) software^55^, as previously described^56–58^. The topology files for all ligands were generated using the SwissParam program^59^. All simulations were carried out in cubic water boxes with a 10 Å thicknesses. The systems were solvated with the TIP3P water model. The overall negative charge of the system was neutralized by adding Na^+^ counter-ions. The initial structures were relaxed through energy minimization by the steepest descent algorithm to avoid steric clashes or inappropriate geometry before the simulation. The number of steps were restricted to 10000, and the maximum force used for the minimization was less than 1000 kJ/mol. A two-step equilibration was performed after the energy minimization. The first step of the equilibration was conducted under an NVT ensemble (constant number of particles, volume, and temperature) for 200 ps at 300 K using a V-rescale thermostat. The second step of equilibration was conducted under an NPT ensemble for 200 ps, where the number of particles, pressure, and temperature were kept constant. The density of the system was stabilized by maintaining the pressure of the system at 1 bar using Parrinello-Rahman barostat. The protein backbone was restrained, while the solvent molecules along with counter-ions were allowed to move during the equilibration process. The equilibrated structures from NPT were used to perform 20 ns MD simulations at 300 K and 1 bar. The LINCS algorithm was used for the bond constraints. The Particle Mesh Ewald (PME) method was used to calculate the long-range electrostatic interactions. The cut-off values of 9 Å and 14 Å were applied to measure the short-range interactions and van der Waals interactions, respectively. The MD simulations were performed under periodic boundary conditions in all directions. The results were analysed using the DS, GROMACS, and visual molecular dynamics (VMD) software.

## Electronic supplementary material

Supplementary information
