# Supplementary material for: New compounds identified through in silico approaches reduce the α-synuclein expression by inhibiting prolyl oligopeptidase in vitro
Source: Sci Rep. 2017 Sep 7;7:10827. doi: 10.1038/s41598-017-11302-0 (PMC5589771; doi:10.1038/s41598-017-11302-0)
Supplement: Supplementary file 1 — Supplementary information [file 41598_2017_11302_MOESM1_ESM.pdf]

New compounds identified through *in silico* approaches reduce the α-synuclein expression by inhibiting prolyl oligopeptidase *in vitro*

Raj Kumar[+], Rohit Bavi[+], Min Gi Jo, Venkatesh Arulalapperumal, Ayoung Baek, Shailima Rampogu, Myeong Ok Kim, Keun Woo Lee*

Division of Applied Life Science (BK21 Plus), Systems and Synthetic Agrobiotech Center (SSAC), Plant Molecular Biology and Biotechnology Research Center (PMBBRC), Research Institute of Natural Science (RINS), Gyeongsang National University (GNU), 501 Jinju-daero, Jinju 52828, Republic of Korea.

*Correspondence should be made to: K.W.L. (kwlee@gnu.ac.kr)

+ These authors contributed equally to this work

Supplementary Table S1. Comparison of fit values of 8 training set compounds mapped with Pharm-A and Pharm-B.

No.	Name	Fit value	
		Pharm-A	Pharm-B
1	Z-321	4.99	4.65
2	SUAM-1221	4.84	3.77
3	UAMC-00021	4.53	4.29
4	JTP-4819	4.08	4.2019
5	ZPP	3.87	4.38
6	Y-29794	3.74	3.59
7	S-17092	2.84	4.01
8	ONO-1603	0.76	3.84

Supplementary Table S2. List of literature-derived active POP inhibitors (IC_{50} < 100 nM) used for pharmacophore validation by Güner-Henry scoring method.

No.	SMILES	IC_{50} (nM)
1	O=C[C@@H]1CCCN1C(=O)[C@@H]2CCCN2C(=O)CC3Cc4ccccc4C3	0.42
2	O=C[C@@H]1CCCN1C(=O)[C@@H]2CCCN2C(=O)C[C@H]3CCc4ccccc4C3	0.45
3	O=C[C@@H]1CCCN1C(=O)[C@@H]2CSCN2C(=O)C[C@H]3CCc4ccccc4C3	0.46
4	O=C(C[C@H]1CCc2ccccc2C1)N3CCC[C@H]3C(=O)N4CCC[C@H]4C#N	0.52
5	O=C(C[C@H]1CCc2ccccc2C1)N3CSC[C@H]3C(=O)N4CCC[C@H]4C#N	0.55
6	O=C[C@@H]1CSCN1C(=O)[C@@H]2CSCN2C(=O)C[C@H]3CCc4ccccc4C3	0.85
7	O=C[C@@H]1CCCN1C(=O)[C@@H]2CSCN2C(=O)CC3Cc4ccccc4C3	1.00
8	O=C(CC1Cc2ccccc2C1)N3CSC[C@H]3C(=O)N4CCC[C@H]4C#N	1.10
9	O=C[C@@H]1CCCN1C(=O)[C@@H]2CCCN2C(=O)OCc3ccccc3	1.10
10	O=C(CC1Cc2ccccc2C1)N3CCC[C@H]3C(=O)N4CCC[C@H]4C#N	1.20
11	O=C(C[C@H]1CCc2ccccc2C1)N3CSC[C@H]3C(=O)N4CCSC4	2.30
12	O=C(C[C@H]1CCc2ccccc2C1)N3CCC[C@@H]3C(=O)N4CCSC4	3.40
13	O=C(C[C@H]1CCc2ccccc2C1)N3CSC[C@H]3C(=O)N4CCCC4	4.00
14	O=C([C@H]1C[C@@H]1c2cccs2)N3[C@@H]4CC[C@@H](CC4)[C@H]3C(=O)N5CCCC5	4.80
15	O=C(OCc1ccccc1)N2CCC[C@H]2C(=O)N3CCC[C@H]3C(=O)c4nc5ccccc5s4	5.00
16	FC(F)(F)[C@H](CCCC(=O)N1[C@H]2CCCC[C@H]2C[C@H]1C(=O)N3CCCC3)c4ccccc4	5.00
17	O=C(C[C@H]1CCc2ccccc2C1)N3CCC[C@@H]3C(=O)N4CCCC4	5.40
18	CC(C)(C)OC(=O)N1[C@@H]2CC[C@@H](C2)[C@@H]1C(=O)N3CCCC3	5.50
19	O=C(CCCc1ccccc1)N2CCC[C@@H]2C(=O)N3CCC[C@H]3C#N	6.00

20	O=C(CC1Cc2ccccc2C1)N3CSC[C@H]3C(=O)N4CCSC4	6.50
21	O=C(CC1Cc2ccccc2C1)N3CCC[C@@H]3C(=O)N4CCSC4	8.70
22	O=C(CCCC(C1CC1)C2CC2)N3[C@@H]4CC[C@@H](CC4)[C@H]3C(=O)N5CCCC5	9.00
23	CCOC(=O)C(=O)[C@@H]1CCCN1C(=O)[C@@H]2CCCN2C(=O)CCCc3ccccc3	10
24	O=C(CC1Cc2ccccc2C1)N3CSC[C@H]3C(=O)N4CCCC4	10
25	OB(O)[C@@H]1CCCN1C(=O)CNC(=O)c2ccnc3ccccc23	11
26	O=C([C@@H]1C[C@H]1c2ccccc2)N3[C@H]4CC[C@H](C4)[C@H]3C(=O)N5CCCC5	14
27	O=C(CC1Cc2ccccc2C1)N3CCC[C@@H]3C(=O)N4CCCC4	17
28	O=C(CCCC(C1CC1)C2CC2)N3[C@H]4CCCC[C@H]4C[C@H]3C(=O)N5CCCC5	17
29	O=C(C[C@H]1CCc2sccc2C1)N3CSC[C@H]3C(=O)N4CCCC4	18
30	O=C(CCCC=C(C1CC1)C2CC2)N3[C@@H]4CC[C@@H](CC4)[C@H]3C(=O)N5CCCC5	20
31	CC(C)(C)OC(=O)N1[C@@H]2CC[C@@H](CC2)[C@@H]1C(=O)N3CC=CC3	20
32	CC(C)(C)OC(=O)N1[C@@H]2CC[C@@H](CC2)[C@@H]1C(=O)N3CCCC3	23
33	O=C(C[C@@H]1CCc2ccccc2C1)N3CSC[C@H]3C(=O)N4CCSC4	24
34	O=C(CC\C=C\C(C1CC1)C2CC2)N3[C@@H]4CC[C@@H](CC4)[C@H]3C(=O)N5CCCC5	24
35	CC(C)(C)OC(=O)N1[C@@H]2CC[C@@H](CC2)[C@@H]1C(=O)N3CCSC3	24
36	O=C(CCCC(C1CC1)C2CC2)N3[C@H]4CC[C@H](C4)[C@H]3C(=O)N5CCCC5	26
37	O=C[C@@H]1CCCCN1C(=O)[C@@H]2CCCN2C(=O)OCc3ccccc3	26
38	O=C(CCCc1ccccc1)N2[C@@H]3CC[C@@H](C3)[C@@H]2C(=O)N4CCCC4	27
39	CC(C)(C)OC(=O)N1[C@H]2CCCC[C@H]2C[C@H]1C(=O)N3CCC3	30
40	CC(C)(C)OC(=O)N1CCC[C@H]1C(=O)N2CCCC2	32
41	O=C(CCCc1ccccc1)N2[C@H]3CCCC[C@H]3C[C@@H]2C(=O)N4CCC	34

	C4	
42	O=C(CCC1Cc2ccccc2C1)N3CSC[C@H]3C(=O)N4CCCC4	36
43	FC(F)(F)[C@H](CC(=O)N1[C@H]2CCCC[C@H]2C[C@H]1C(=O)N3CCCC3)c4ccccc4	36
44	O=C(C[C@@H]1CCc2ccccc2C1)N3CSC[C@H]3C(=O)N4CCCC4	38
45	O=C(CCCCC(C1CC1)C2CC2)N3[C@@H]4CC[C@@H](CC4)[C@H]3C(=O)N5CCCC5	40
46	O=C(C[C@@H]1CCc2ccccc2C1)N3CCC[C@@H]3C(=O)N4CCSC4	40
47	C[C@H](NC(=O)c1ccnc2ccccc12)C(=O)N3CCC[C@H]3C#N	44
48	FC(F)(F)[C@H](CCCC(=O)N1[C@H]2CCCC[C@H]2C[C@H]1C(=O)N3CCCC3)C4CCCC4	46
49	O=C(C[C@@H]1CCc2ccccc2C1)N3CCC[C@@H]3C(=O)N4CCCC4	48
50	O=C([C@H]1C[C@@H]1c2ccccc2)N3[C@@H]4CC[C@@H](CC4)[C@H]3C(=O)N5CCCC5	50
51	O=C(CCCc1ccccc1)N2[C@@H]3CC[C@@H](CC3)[C@@H]2C(=O)N4CCCC4	50
52	O=C(CCC(C1CC1)C2CC2)N3[C@H]4CCCC[C@H]4C[C@H]3C(=O)N5CCCC5	58
53	O=C(CCCC1Cc2ccccc2C1)N3CSC[C@H]3C(=O)N4CCCC4	60
54	O=C(CC1=Cc2ccccc2C1)N3CCC[C@@H]3C(=O)N4CCCC4	69
55	O=C([C@H]1C[C@@H]1c2ccccc2)N3[C@H]4CC[C@H](C4)[C@H]3C(=O)N5CCCC5	78
56	O=C(CCSC(C1CC1)C2CC2)N3[C@H]4CCCC[C@H]4C[C@H]3C(=O)N5CCCC5	86
57	O=C(OCc1ccccc1)N2CCC[C@H]2C(=O)N3CCCC3	86

Supplementary Table S3. List of potential virtual hit compounds obtained from the docking-based virtual screening process.

Name	Goldscore	Chemscore
Compound 1 (Hit 1)	74.34	-34.68
Compound 2	69.93	-34.18
Compound 3	69.73	-32.89
Compound 4 (Hit 2)	69.64	-33.69
Compound 5	67.37	-37.28
Compound 6	66.55	-36.51
Compound 7	64.61	-36.14
Compound 8	64.51	-32.52
Compound 9	63.88	-30.84
Compound 10	63.78	-33.88
Compound 11	62.44	-38.06
Compound 12	62.19	-33.24
Compound 13	61.56	-29.98
Compound 14	61.43	-27.04
Compound 15	60.38	-31.47
Compound 16	60.26	-34.00
Compound 17	59.36	-26.21
Compound 18	58.11	-35.61
Compound 19	57.30	-28.22
Compound 20	57.14	-34.46
Inh 1	67.98	-26.12
Inh 2	56.89	-25.78

Supplementary Table S4. The specifications of systems used for molecular dynamics simulations of compounds with POP.

No.	System	No. of TIP3P water molecules	No. of Na+ counter ions	System size (nm)
1	POP + Inh 1 (Z-321)	33127	22	7.753 × 5.957 × 8.700
2	POP + Inh 2 (S-17092)	33125	22	7.753 × 5.957 × 8.700
3	POP + Hit 1	33124	22	7.753 × 5.957 × 8.700
6	POP + Hit 2	33117	22	7.753 × 5.957 × 8.700

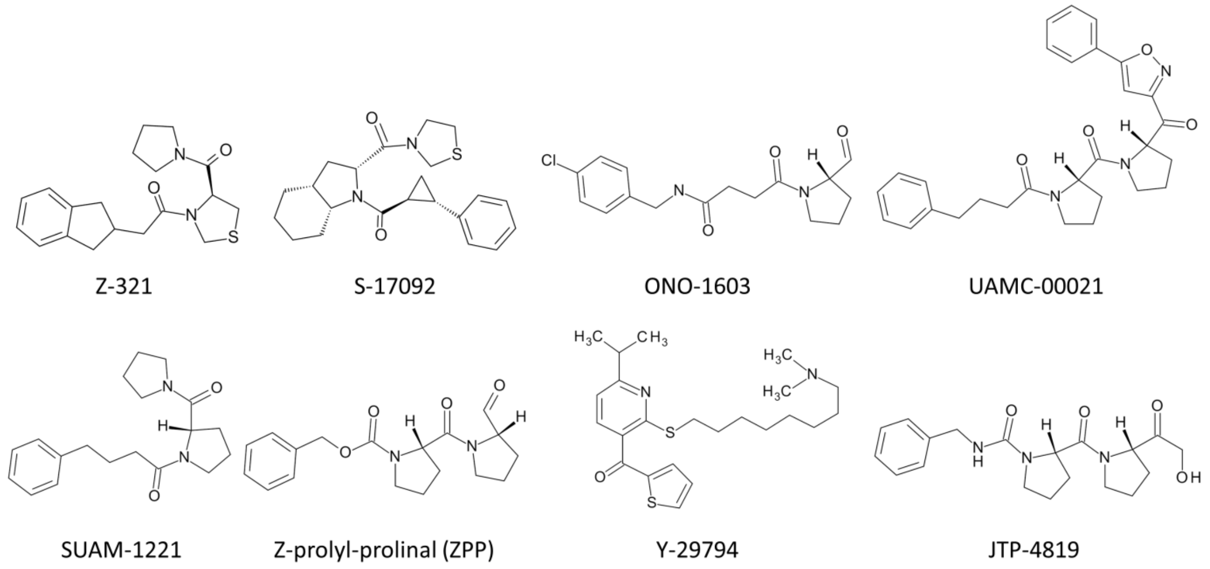

Supplementary Figure S1. Training set compounds used in common-feature pharmacophore generation.

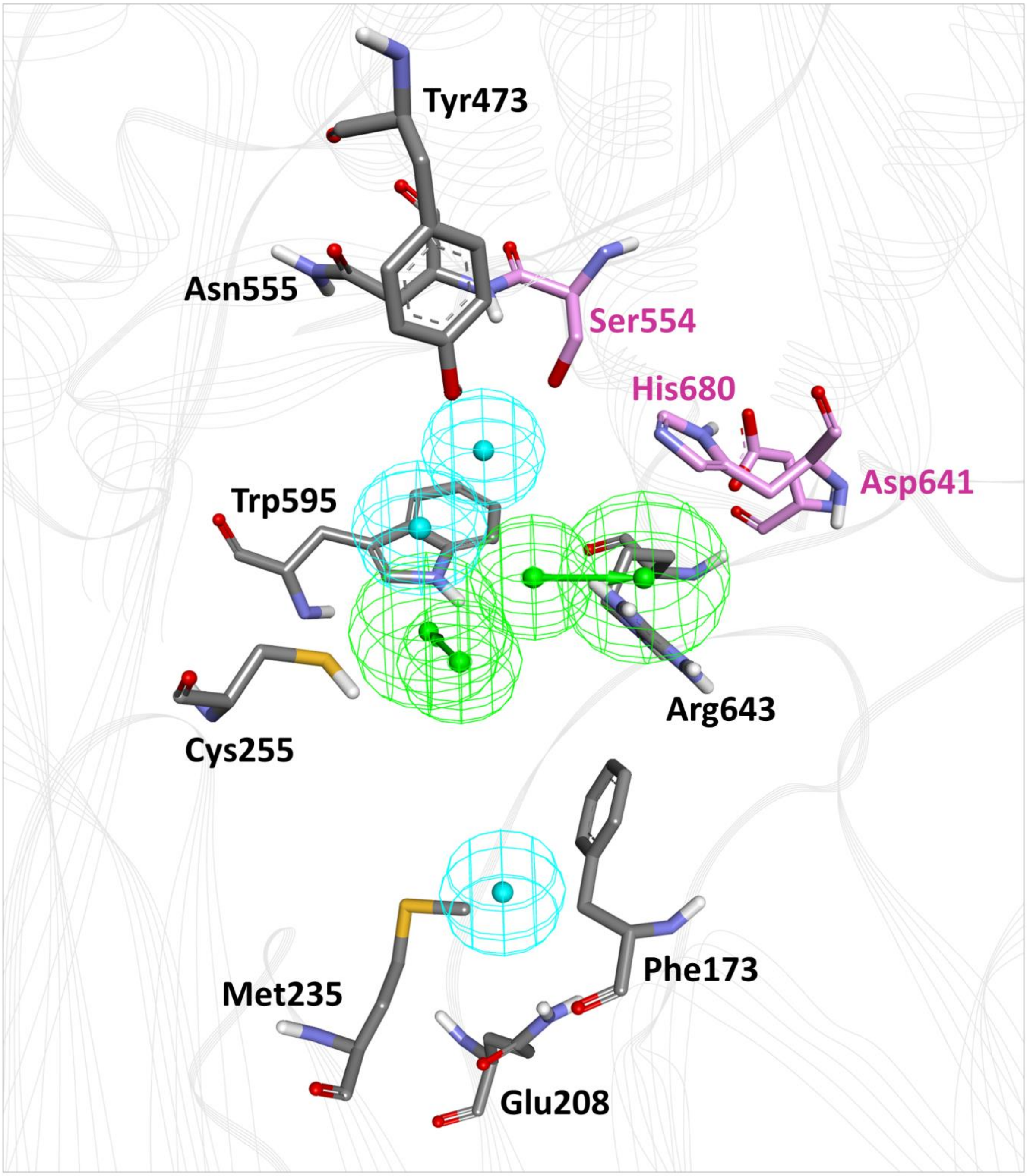

Supplementary Figure S2. Ligand-based pharmacophore model, Pharm-A overlaid onto the active site of POP (PDB ID: 3DDU).

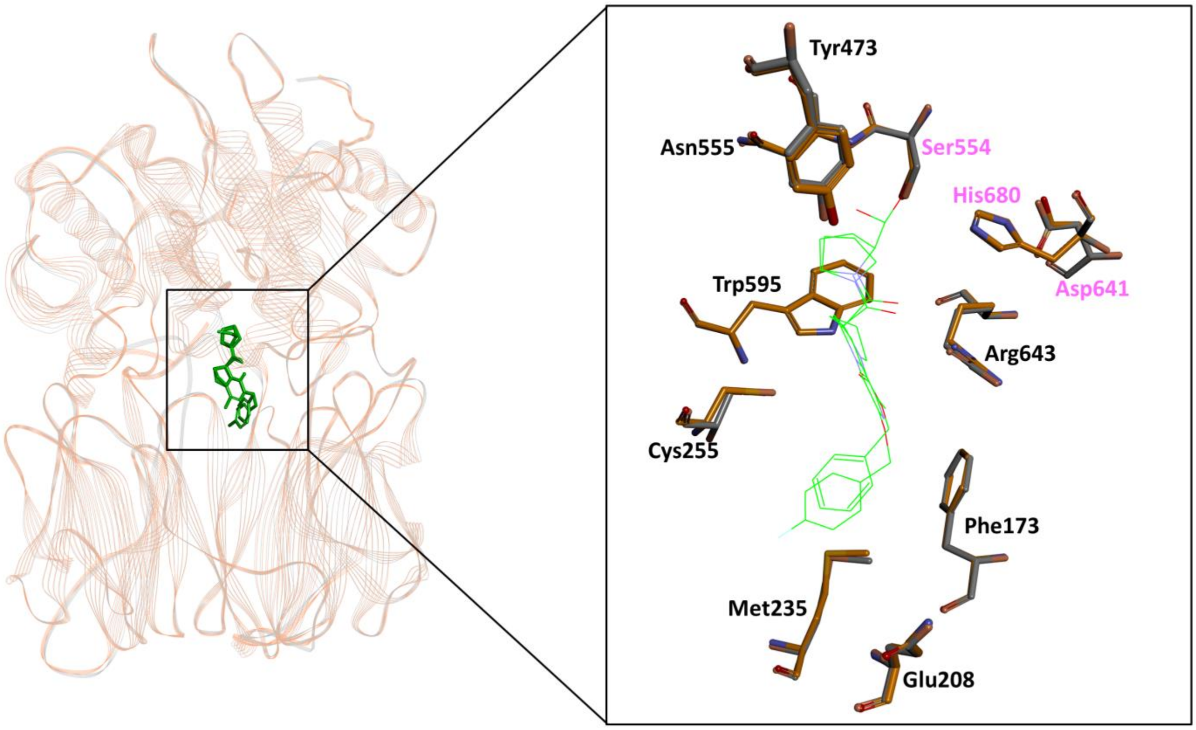

Supplementary Figure S3. Superimposition of human POP (Grey, PDB ID: 3DDU) with porcine POP (Orange, PDB ID: 1QFS) showing high conservation of catalytic residues at the active site (enlarged). The active site residues were represented as the stick model (catalytic residues in pink) while the inhibitors were shown as lines in green color.

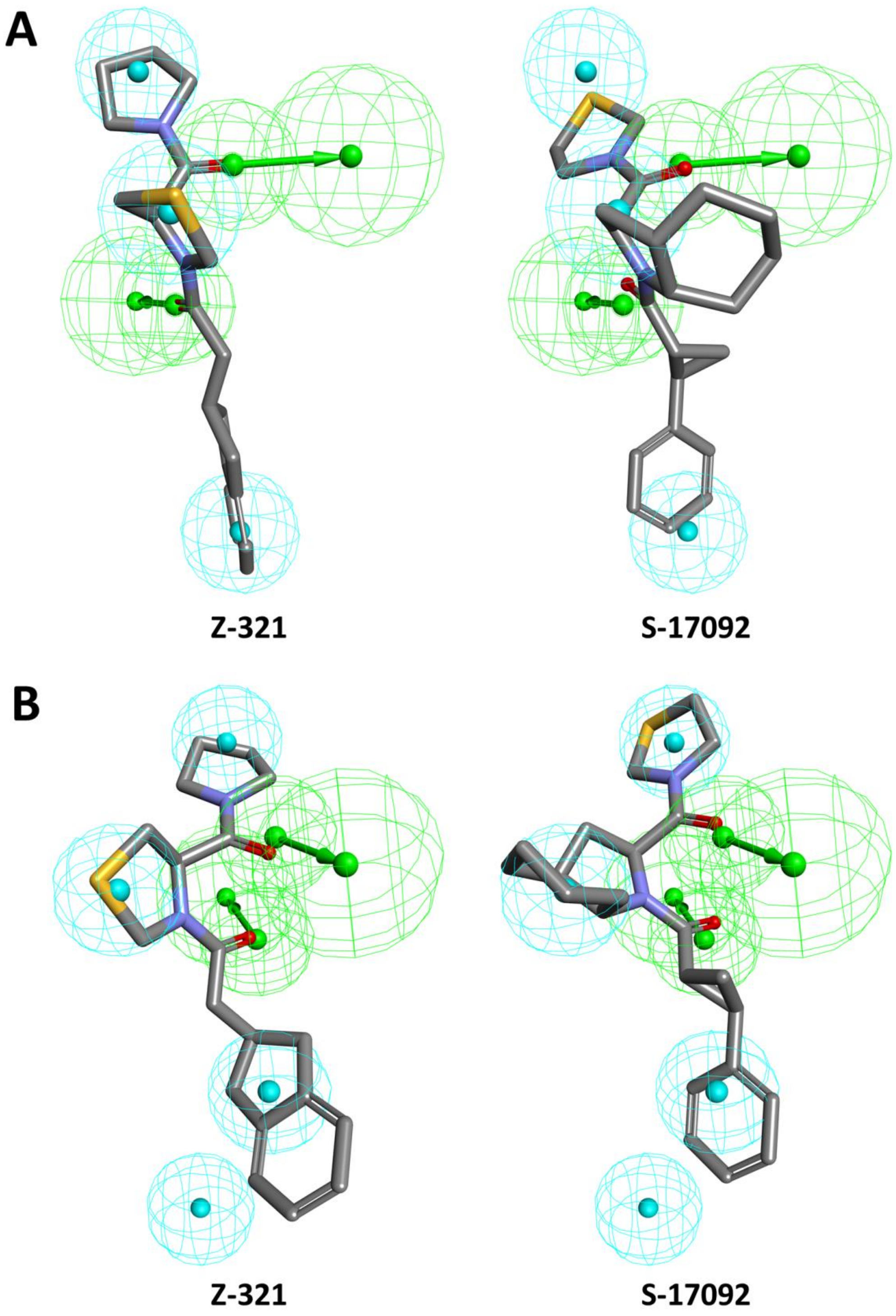

Supplementary Figure S4. Mapping of reference inhibitors Z-321 and S-17092 onto pharmacophore models (A) Pharm-A, and (B) Pharm-B.

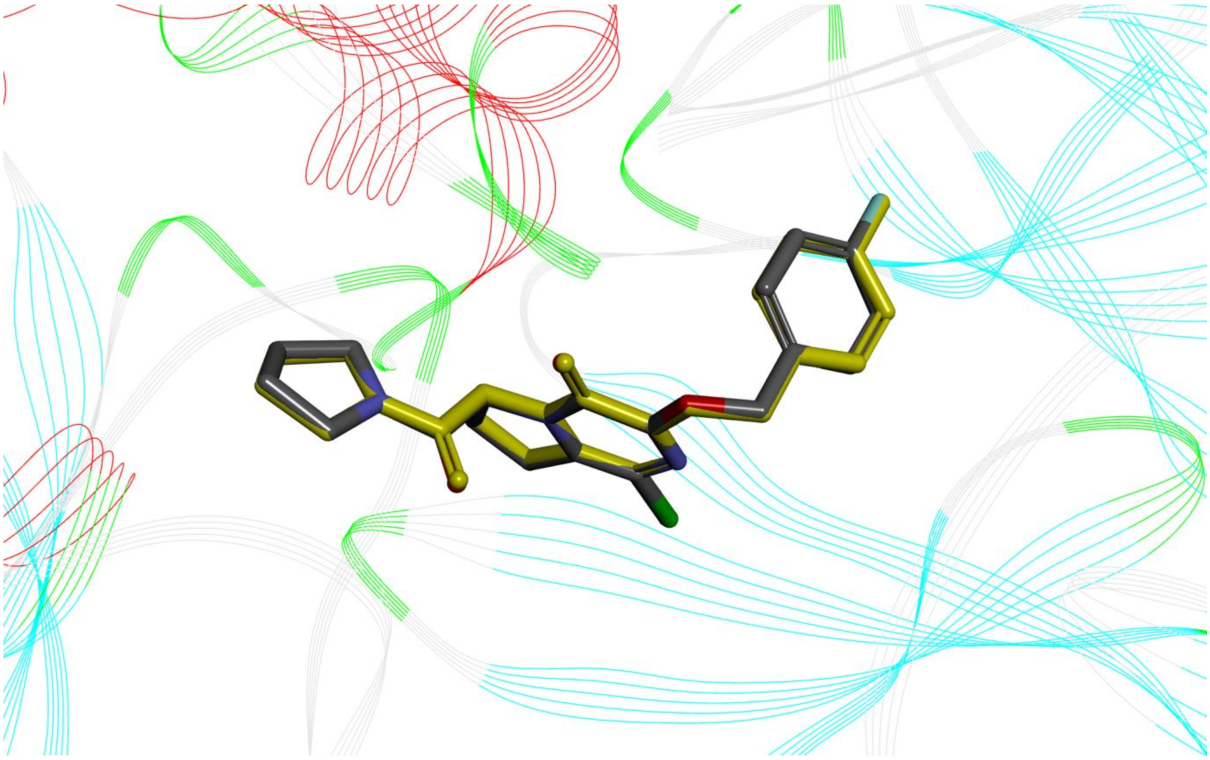

Supplementary Figure S5. Structure overlap between co-crystal (Gray; PDB ID: 3DDU) and its docked orientation (yellow).

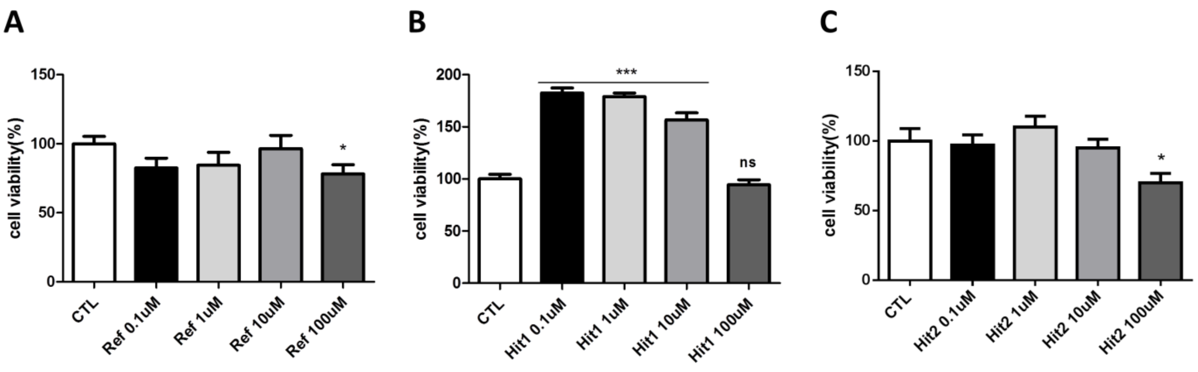

Supplementary Figure S6. Comparative analysis of reference inhibitor, Hit 1 and Hit 2 on cell viability and cytotoxicity in SH-SY5Y human neuroblastoma cells. SH-SY5Y cells were incubated for 24h with (A) Ref, (B) Hit 1, and (C) Hit 2 compounds at 0.1, 1, 10, and 100μM concentration. The cell viability and cytotoxicity was detected by MTT assay. The data is shown as the mean ± SEM of triplicate for *in vitro* three independent experiments. The histogram represent percentage change (%) versus control group; * p<0.05, *** p<0.001, ns p>0.05.

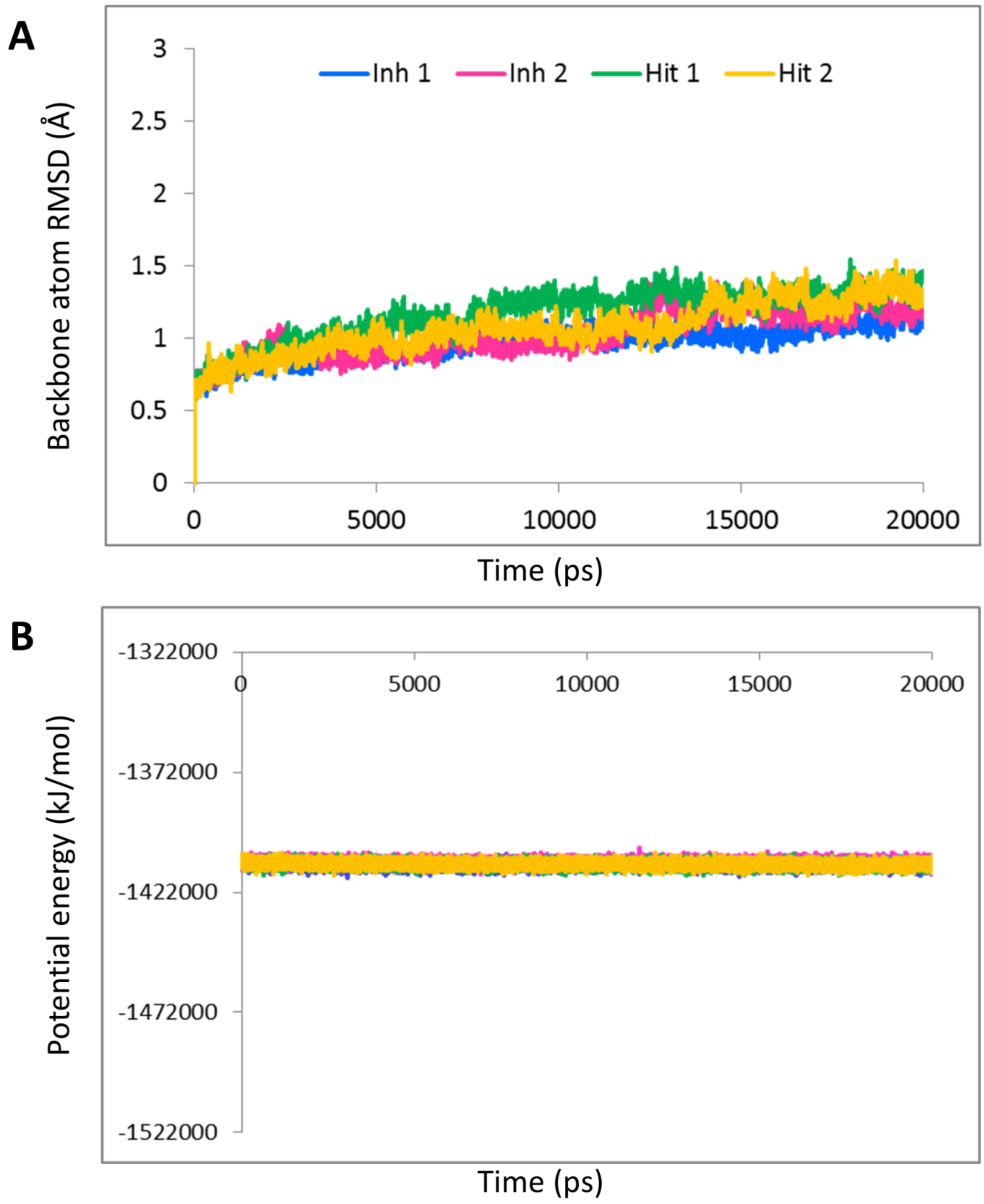

Supplementary Figure S7. The representation of the RMSD and potential energy plots for six complex systems. (A) The RMSD of the protein backbone atoms. (B) The potential energies of the systems. These plots were calculated during 20 ns MD simulations for each complex. Inh 1 (Z-321), Inh 2 (S-17092), Hit 1, and Hit 2 are represented by the blue, magenta, green, and orange lines, respectively.

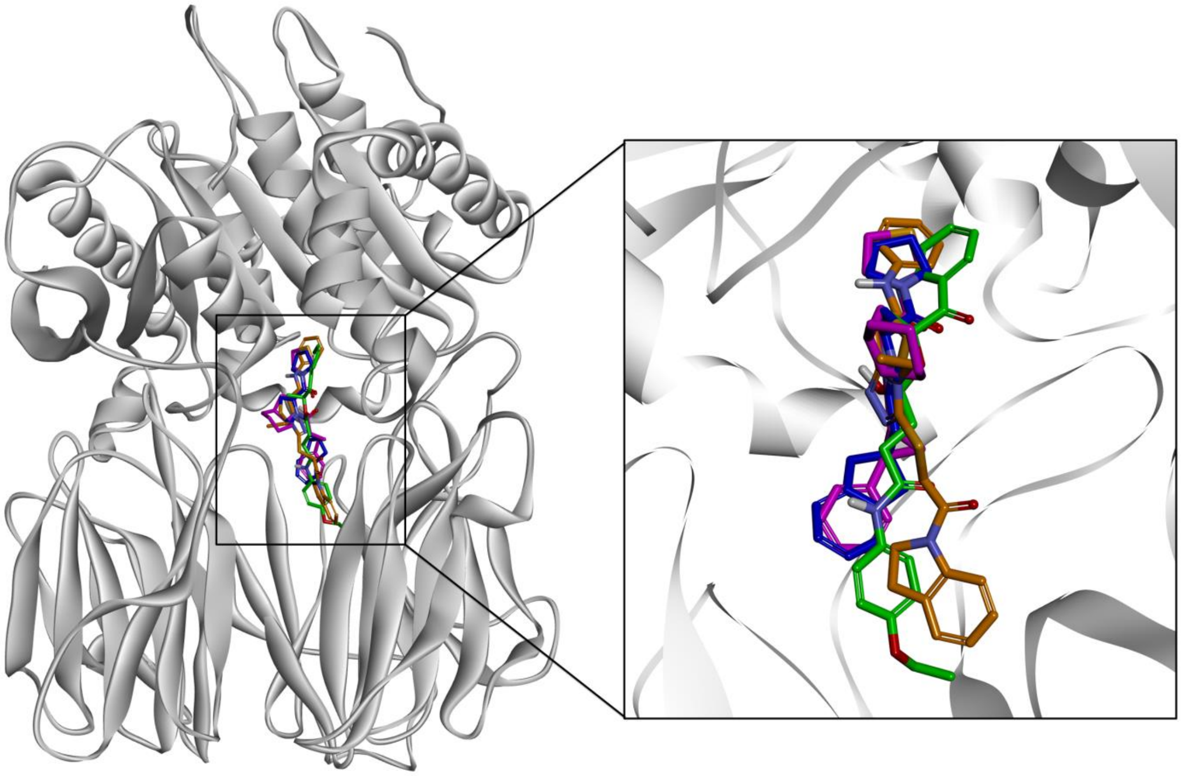

Supplementary Figure S8. The binding orientations of reference and hit compounds in the active site of POP. All compounds in their representative structures were superimposed (left) and enlarged (right). The protein is represented in grey ribbons. Blue, magenta, green, and orange colors represent Inh 1, Inh 2, Hit 1, and Hit 2, respectively.

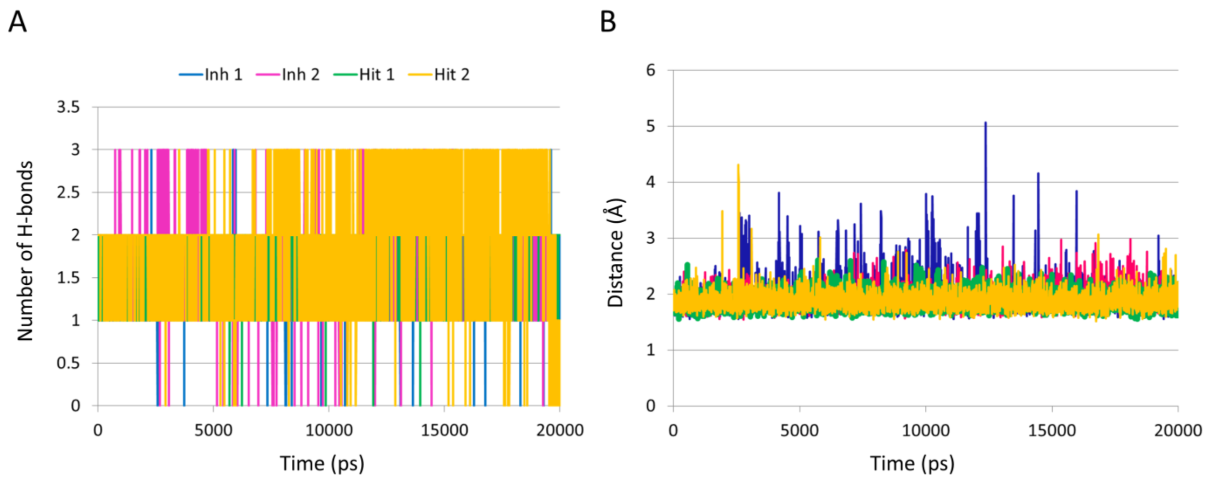

Supplementary Figure S9. Representation of the number of hydrogen bonds between the compounds and protein during the 20 ns MD simulations (A). Calculation of hydrogen bond distance between Arg643 and the compounds during the simulations (B). Inh 1, Inh 2, Hit 1, and Hit 2 are represented by the blue, magenta, green, and orange lines, respectively.

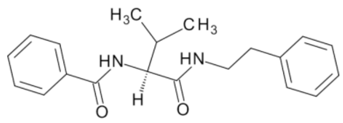

Compound 2

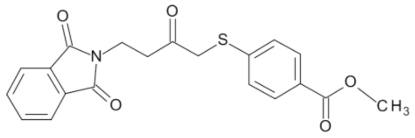

Compound 3

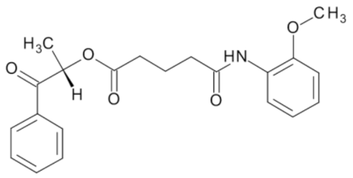

Compound 5

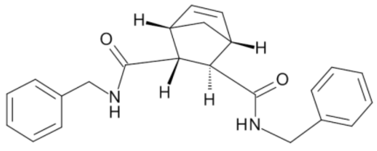

Compound 6

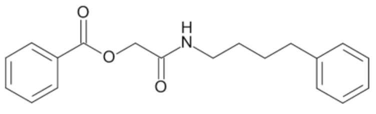

Compound 7

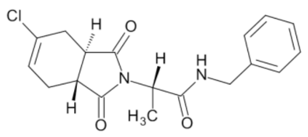

Compound 8

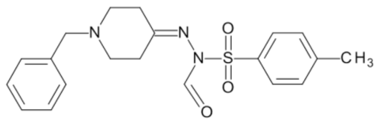

Compound 9

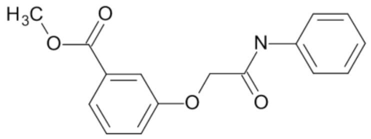

Compound 10

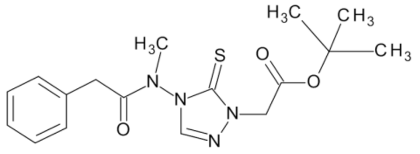

Compound 11

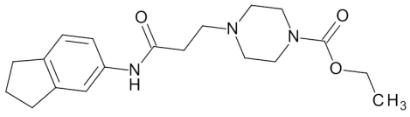

Compound 12

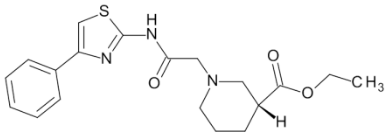

Compound 13

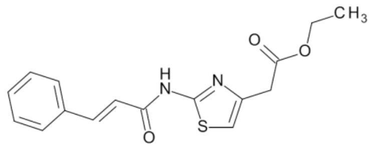

Compound 14

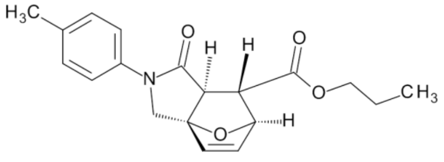

Compound 15

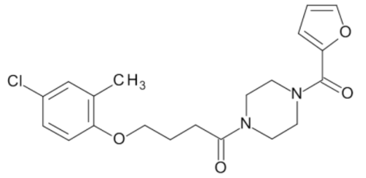

Compound 16

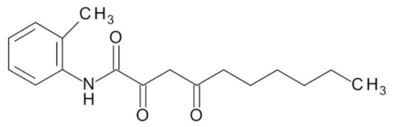

Compound 17

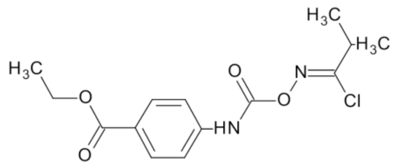

Compound 18

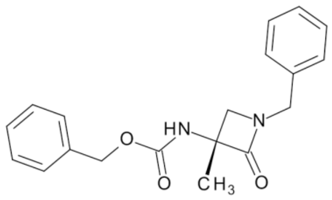

Compound 19

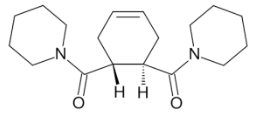

Compound 20

Supplementary Figure S10. Chemical structures of the possible candidates from the virtual screening.
